# Mastitis Control and Intramammary Antimicrobial Stewardship in Ireland: Challenges and Opportunities

**DOI:** 10.3389/fvets.2022.748353

**Published:** 2022-04-11

**Authors:** Simon J. More, Catherine McAloon, Pablo Silva Boloña, Luke O'Grady, Frank O'Sullivan, Michelle McGrath, Willie Buckley, Kevin Downing, Patrick Kelly, Eoin G. Ryan, Finola McCoy

**Affiliations:** ^1^Centre for Veterinary Epidemiology and Risk Analysis, UCD School of Veterinary Medicine, University College Dublin, Belfield, Ireland; ^2^Herd Health and Animal Husbandry, UCD School of Veterinary Medicine, University College Dublin, Belfield, Ireland; ^3^Teagasc, Animal and Grassland Research and Innovation Centre, Fermoy, Ireland; ^4^School of Veterinary Medicine and Science, University of Nottingham, Sutton Bonington Campus, Leicestershire, United Kingdom; ^5^Patrick Farrelly & Partners, Dublin, Ireland; ^6^Animal Health Ireland, Carrick on Shannon, Ireland; ^7^Riverview Vet Clinic, Bandon, Ireland; ^8^Irish Cattle Breeding Federation, Ballincollig, Ireland; ^9^Independent Researcher, Cahir, Ireland

**Keywords:** antibiotic stewardship, mastitis control, prescribing, dairy production, international best-practice, Ireland

## Abstract

The Veterinary Medicines Regulation (EU 2019/6) came into force in all EU member states on 28 January 2022. This regulation places particular emphasis on prudent and responsible antimicrobial use in food animal production. Key changes include restrictions on the prophylactic use of antimicrobials in animals, and the possibility to reserve certain antimicrobials for humans only. The Regulation presents challenges to the Irish dairy industry, particularly with respect to current approaches to dry cow therapy. In response, the CellCheck technical working group (TWG, a technical group working in support of CellCheck, the national mastitis control programme) have developed pragmatic national and farm-level recommendations in support of improved mastitis control and intramammary antimicrobial stewardship in the Irish dairy industry. This paper outlines these recommendations, and provides an overview of the evidence considered to inform the TWG during its work (including the Regulation, policy perspectives, international best-practice, international scientific reviews and specific Irish challenges). In many key areas of concern, the TWG recognises the challenges in seeking to shape recommendations in the absence of robust and practical scientific evidence. For this reason, some of the recommended actions are pragmatic in nature, informed by national and international experiences. Periodic programme review will be needed, informed by ongoing monitoring of key performance indicators, to identify those actions that are most effective in an Irish context.

## Introduction

Antimicrobials targeting bacterial infections are widely used in dairy production, both to treat and prevent intramammary infections in dairy cows. The “five-point plan” of the UK's National Institute for Research in Dairying, in place since the late 1960s to control contagious mastitis on dairy farms, centered around five key recommendations, including routine maintenance of milking machines, post-milking teat disinfection, identification and antimicrobial treatment of clinical cases, whole herd antimicrobial dry-cow therapy (DCT) and the culling of chronically infected cows (1–3). These recommendations were facilitated by the development of persistent antimicrobial formulations (4, 5), both to shorten the duration of infection (following administration at drying off) and to prevent the establishment of new infection (at or following drying off). The plan proved extremely effective in managing contagious pathogens, with the incidence in clinical mastitis falling from in excess of 150 cases per 100 cows per year in some herds to <40 cases per 100 cows per year between the late 1960s and early 1980s (6). During a similar time period, national average bulk milk somatic cell counts (BMSCC) also dropped from over 600,000 cells/mL to around 400,000 cells/mL (6). With the emergence of new patterns of disease, particularly the rise in environmental pathogens, there has been a need for some adaptation to the five-point plan in recent years (7).

There is increasing focus on the phenomenon of antimicrobial resistance (AMR), that is, the emergence of bacteria that do not respond to antimicrobial treatment (8–10), with AMR now considered one of the most important global threats to human and animal health (11). In response, there are international efforts to limit antimicrobial use in human and veterinary medicine, including food animal production, with a particular focus on the excessive or inappropriate use of antimicrobial agents.

In force in all EU member states from 28 January 2022, the Veterinary Medicines Regulation (Regulation (EU) 2019/6, subsequently referred to as the “Regulation”) (12) places particular emphasis on prudent and responsible antimicrobial use in food animal production. Key changes include restrictions on the prophylactic use of antimicrobials in animals, and the possibility to reserve certain antimicrobials for humans only. As a consequence, blanket DCT, in which all cows are routinely treated with antimicrobials at drying off regardless of their infection status (13), is no longer acceptable. Rather, EU farmers are required to move toward selective DCT, whereby only animals with evidence of infection at drying off should receive an antimicrobial.

In Ireland, the national mastitis control programme, known as CellCheck, was established in late 2010 (14). The programme is managed by Animal Health Ireland, and delivered in partnership with industry, government and service providers. Technical aspects of the programme are guided by the CellCheck technical working group (TWG) (15), which is a group of approximately 18 experts who meet regularly to discuss and agree technical issues in support of the programme. TWG members are drawn from a range of relevant disciplines and are appointed in their individual capacity independent of their organizations of employment (16).

The Regulation presents challenges to the Irish dairy industry, particularly with respect to current approaches to DCT. In response, the CellCheck TWG have developed pragmatic national and farm-level recommendations in support of improved mastitis control and intramammary antimicrobial stewardship in the Irish dairy industry. This paper outlines these recommendations, and provides an overview of the evidence considered to inform the TWG during its work (including the Regulation, policy perspectives, international best-practice, international scientific reviews and specific Irish challenges).

## Materials and Methods

The CellCheck TWG met regularly over a number of years, seeking a detailed understanding of the factors that support and constrain improved mastitis control and intramammary antimicrobial stewardship in the Irish dairy industry. The TWG members represented a range of technical disciplines relevant to dairy production, including animal and dairy science, data science, epidemiology and veterinary medicine. The TWG members each had a detailed understanding of the Irish dairy industry, in their roles in academia, farm advisory services or veterinary practice.

Aspects of the Regulation relevant to intramammary antimicrobial prescribing and use were reviewed, and the regulatory implications, particularly for herds with suboptimal mastitis control, were considered. A broad range of evidence sources were reviewed in support of these discussions, including international, national and Irish policy perspectives, comparison with international best-practice and international scientific reviews relevant to mastitis control and antimicrobial stewardship. The key challenges in Ireland relating to mastitis control and intramammary antimicrobial stewardship were distilled. This work was undertaken through narrative desk-based reviews and based on the knowledge and experience of TWG members. National and farm-level recommendations were agreed on the basis of consensus, seeking actions that were pragmatic and likely effective based on the evidence available.

## The Veterinary Medicines Regulation

### The Regulation

The Regulation has been in force in all EU member states from 28 January 2022. The key objectives of the legislation include the promotion of prudent and responsible antimicrobial use to minimise AMR in animals and prevent the spread of antimicrobial-resistant bacteria into the food chain, to promote the availability of veterinary medicinal products through innovation and competition, and to establish a modern fit-for-purpose legal framework.

Relevant to dairy production, key changes with this Regulation include:

- Restrictions on the prophylactic use of antimicrobials in animals, so that they may only be used in exceptional cases, in an individual or a restricted number of animals, when the risk of infection is very high and the consequences are likely to be severe
- Restriction on the metaphylactic use of antimicrobials in groups of animals
- The possibility to reserve certain antimicrobials for humans only
- The need for member states to collect data on the sale and use of antimicrobials at prescriber and user level (12).

### The Therapeutic, Metaphylactic and Prophylactic Use of Intramammary Antimicrobials

In general terms, antimicrobials are used in three specific contexts:

- Therapeutic use, which refers to treatment given to animals with evidence of infection of the mammary gland. This could include clinical evidence of infection (such as a clinical presentation of a swollen, hot quarter or changes in the milk consistent with clinical mastitis) or evidence of subclinical infection based on either direct (culture or polymerase chain reaction, PCR) or indirect (individual cow somatic cell count, SCC) information (17).
- Metaphylactic use, which is defined in EU Regulation 2019/6 as “*the administration of a medicinal product to a group of animals after a diagnosis of clinical disease [bacterial infection] in part of the group has been established, with the aim of treating the clinically sick [infected] animals and controlling the spread of the disease to animals in close contact and at risk and [treating animals] which may already be subclinically infected”*.
- Prophylactic use (preventive treatment) is defined in EU Regulation 2019/6 as “*the administration of a medicinal product to an animal or group of animals before clinical signs of a disease, in order to prevent the occurrence of disease or infection”*.

With respect to intramammary antimicrobials, treatment during lactation is primarily only administered to animals with evidence of clinical infection, and is therefore deemed therapeutic use (17). Metaphylactic use is rarely necessary during lactation, and only considered in response to large-scale outbreaks of highly contagious mastitis, which is a rare event and generally related to suboptimal hygiene, milking routine or farm management. At drying off, traditional blanket DCT (the routine treatment of all animals with antimicrobial therapy) can either be classified as therapeutic or prophylactic usage, depending on the cow's true infection status (17). It has been suggested that blanket DCT can also constitute metaphylactic usage, as the true infection status is not always known. However, this term is not appropriate for DCT because the milking process has ceased and thus the primary risk factor for the spread of contagious mastitis has been removed.

### Antimicrobials Reserved for Human Use

The Antimicrobial Advice Ad Hoc Expert Group (AMEG) of the European Medicines Agency has categorised antimicrobials based on the potential consequences to public health of increased antimicrobial resistance when used in animals and the need for their use in veterinary medicine (18). The EMA categorization is informed by other work to classify antimicrobials, including that adopted by the WHO for human health, where the categories used include Important Antimicrobials (IAs), Highly Important Antimicrobials (HIAs) and Critically Important Antimicrobials (CIAs, with further prioritisation of Highest Priority CIAs, HP-CIAs) (19).

The AMEG categorization is intended as a tool to support decision-making by veterinarians within the European Union, and includes key action words (avoid, restrict, caution, prudence) attributed to each category:

- *Category A (“Avoid”)*, includes antimicrobials that are currently not authorised in veterinary medicine in the EU. These classes should not be used in food-producing animals, and may be given to individual companion animals only under exceptional circumstances.
- *Category B (“Restrict”)* includes classes that are critically important in human medicine and use in animals should be restricted to mitigate the risk to public health. Their use should be considered only when there are no antimicrobials in Categories C or D that could be clinically effective, and should be based on antimicrobial susceptibility testing where possible.
- Category C *(“Caution”)*, includes classes where there are alternatives for human medicine. Should only be considered when there are no antimicrobials in Category D that could be clinically effective.
- Category D *(“Prudence”)*, should be used as first-line treatments where possible, and should be used prudently, only when medically needed (20).

### Regulatory Implications, Particularly in Herds With Suboptimal Mastitis Control

In the context of the Regulation, several factors will influence intramammary antimicrobial prescribing, particularly in herds with suboptimal mastitis control:

- There will be no direct change to the ongoing need for therapeutic usage of antimicrobials, either in-lactation or at drying-off. As outlined in the Regulation, a veterinary prescription “*shall only be issued after a diagnosis of the infectious disease by a veterinarian”* and on foot of “*a clinical examination or any other proper assessment of the health status of the animal or group of animals by a veterinarian”* (12).
- The Regulation explicitly states that antimicrobials “*shall not be applied routinely nor used to compensate for poor hygiene, inadequate animal husbandry or lack of care or to compensate for poor farm management”* (12). Consequently, there is an imperative that action is taken to resolve suboptimal management conditions that contribute to increased infectious challenge at all stages of production, particularly during milking but also during the dry period and around calving.
- Animal-level information will be required to guide decision-making, specifically to distinguish infected and non-infected animals. In all herds, this will be challenging given the imperfect operating characteristics of available diagnostic tests. There are heightened challenges in herds with suboptimal mastitis control, noting that the negative predictive value (the probability that a test negative individual is truly non-infected) falls as prevalence increases (21). In these herds, there is greater uncertainty about the infection status of cows classified as non-infected, and consequently a shift from blanket to selective DCT would be associated with greater inherent risk for these animals, with the potential that infected animals will not be treated. Prescribers need to be aware that the welfare of cows may be compromised if they are infected but not treated.
- Antimicrobial cure rates during lactation are frequently disappointing, and considerable reliance may be placed on DCT to resolve infection (17). In herds with suboptimal mastitis control, this required shift away from prophylactic antimicrobial usage at drying-off will make herd-level control more challenging, given the reduced opportunities during the dry period for cure (of untreated, infected cows) and prevention (of untreated, uninfected cows). The latter can be mitigated with teat sealants when applied correctly (22).
- The use of teat sealant without an antimicrobial will adversely impact cow health and welfare if aseptic technique at insertion is not carefully followed (23). This may be particularly challenging in situations of poor farm management.

## The Evidence Considered

### Policy Perspectives

#### International Policies

A global action plan on antimicrobial resistance was adopted by the World Health Organization (WHO) in 2015 (24), with support from the Food and Agriculture Organization of the United Nations (FAO) (25) and the World Organization for Animal Health (OIE) (26), with responsible and prudent use of these medicines in human and animal health as a key goal. The OIE has outlined key strategies for prudent use of antimicrobials in support of these global efforts, including improved AMR awareness and understanding, strengthened knowledge through surveillance and research, the support of good governance and capacity building, and encouragement for implementation of international standards (27). The WHO has identified criteria for classifying antimicrobials of importance to human medicine (19) and similar guidelines have been produced for food producing animals (28).

There is international acceptance of the need for a One Health approach given the substantial use of antimicrobial agents in both human medicine and food animal production. This approach also ensures coordination across all relevant sectors, both to reduce antimicrobial usage (10, 29) and to limit the emergence and spread of AMR (30–32). Based on findings from a recent systematic review, reducing the level of antimicrobial use in livestock populations is likely to be a beneficial strategy for both animals and humans (33). Further, there are examples of linkages between AMR in food animals and humans, through the acquisition of resistant bacteria or, more importantly, through the spread of resistance genes (34). Although the mechanisms for cross-species transmission of resistant bacteria and their genetic elements are not fully understood, it seems clear that the health of humans, animals, and the ecosystem are intricately linked, and that an interdisciplinary and multi-sectoral approach will be required to address the problem of AMR (10).

There is a particular focus on antimicrobial stewardship, which refers to the efforts made to ensure that antimicrobials are used only when necessary and appropriate. It was first established as a set of “responsible use” policy measures to combat AMR in human hospitals (35) and is now used widely in human medicine (36, 37). In food animal production, antimicrobial stewardship similarly refers to a commitment to judicious use of antimicrobials (38–40), including efforts to limit inappropriate usage, to optimise the choice, dose rate, route, and duration of therapy to maximise clinical cures, and to minimise the emergence and spread of AMR.

#### EU Policies

In Europe, the European Commission developed guidelines on the prudent use of antimicrobials in veterinary medicine in 2015 (41). In 2017, the Commission adopted the EU One Health Action Plan against AMR (42), with key objectives including making the EU a best-practice region, boosting research, development and innovation, and shaping the global agenda. Subsequently, the Farm to Fork Strategy (as one of the policy areas within the European Green Deal) was adopted in 2020 as a tool to help share the EU's path toward sustainable food systems (43). The Farm to Fork Strategy seeks to accelerate the transition within the EU to a sustainable food system, given the linkages between healthy people, healthy societies and a healthy planet. AMR is listed as an area of particular concern within this strategy, with a key target being a 50% reduction in overall EU sales of antimicrobials for farmed animals and for agriculture by 2030. Regulations 2019/4 (on medicated feed) (44) and 2019/6 (12) provide a wide range of measures to fight AMR and promote a more prudent and responsible use of antimicrobials in animals (20).

#### National Policies and Actions

In Ireland, the National Action Plan on Antimicrobial Resistance (termed iNAP) was established in 2017. The plan recognises the urgent and growing problem of antimicrobial resistance for human health worldwide, and aims to implement policies and actions to prevent, monitor and combat AMR across the health, agricultural and environmental sectors (45). iNAP objectives relevant to mastitis control and intramammary antimicrobial stewardship in the dairy industry include:

- Under Strategic objective 2 (enhance surveillance of antibiotic resistance and antibiotic use), to develop and implement a system for the collection of data in relation to usage of intramammary tubes in the dairy sector
- Under Strategic objective 3 (reduce the spread of infection and disease), to implement measures to improve the national Somatic Cell Count through the CellCheck programme, including promoting further uptake of milk recording and of selective dry cow therapy (SDCT) *via* Targeted Advisory Service on Animal Health (TASAH)-funded Dry Cow Consults, and to develop and pilot a delivery mechanism of farm-specific mastitis investigations by appropriately trained local service provider teams.

In the food animal industry, a code of good practice regarding the responsible prescribing and use of antimicrobials has been developed (46). In the dairy industry, Animal Health Ireland coordinates CellCheck, Ireland's national mastitis control programme (47), and have produced guidelines for the use of selective DCT (48) and prudent intramammary antimicrobial prescribing (49).

### Comparison With International Best-Practice

In the following sections relating to antimicrobial usage and stewardship, comparison is primarily made between Denmark, Ireland and the Netherlands. These countries are each required to comply with the same EU legislation, and each have important national dairy industries with a not-dissimilar value and mix of dairy exports. Further, in each country, the quality of dairy product is critical and all supply commodity for the manufacture of infant formula and other high value markets (50). Comparison with Australia is also relevant, in the context of mastitis control.

#### Mastitis Control

The development of the CellCheck programme was substantially informed by Countdown Downunder, the Australian national mastitis control programme (51), and a memorandum of understanding is in place between these two programmes. Established in 1998, the Countdown Downunder programme placed particular emphasis on resource development, including the Countdown Downunder Farm Guidelines for Mastitis Control, in which mastitis control information was arranged according to stage of lactation (calving, lactation, late lactation, drying-off, dry period), a dry cow consult to support decision making at drying off, and a Mastitis Focus Report providing an overview of udder health in an individual herd (52). Training is also critical, leading to skills development among veterinarians, field officers and milk quality staff, herd improvement personnel, milking machine technicians and other dairy advisors. Key features of the programme have included clear, consistent industry-agreed messages, a regional advisory capacity for mastitis control, delivering extension messages through local advisers and using a team approach when dealing with mastitis issues. A 10-year review confirmed initial progress toward industry cell count goals, however, this trend was subsequently reversed as a result of severe environmental conditions (51).

**Figure 1:**
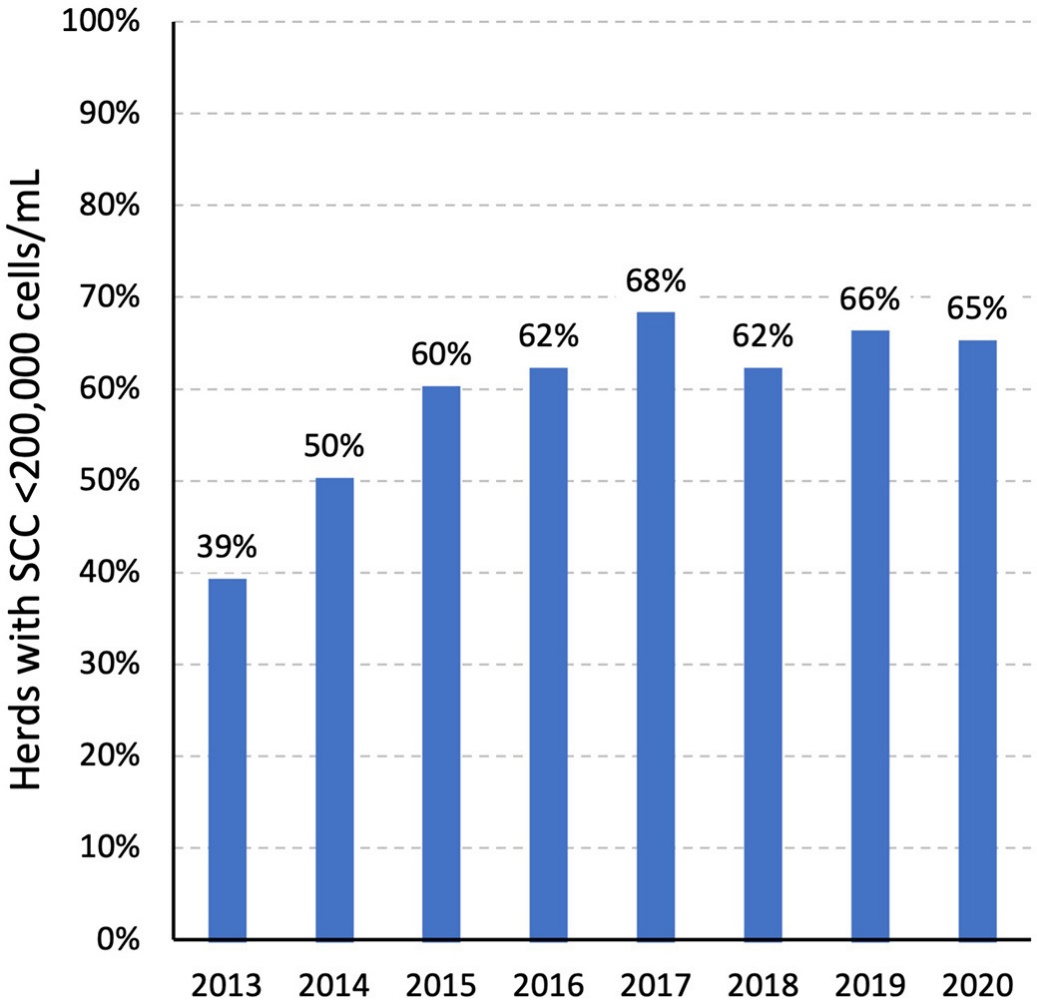
The percentage of Irish dairy herds with an annual unadjusted geometric mean bulk milk SCC <200,000 cells/mL during 2013–20, by year. Source: CellCheck, Animal Health Ireland.

CellCheck follows a similar resource and training model, seeking to increase farmer and advisory awareness of appropriate mastitis control strategies, and to facilitate access to resources to assist with on-farm mastitis control. The programme is informed by the CellCheck TWG, which has guided the development of the Farm Guidelines for Mastitis Control, the CellCheck Dry Cow Strategy, and the Farm Summary Report (which provides an overview of udder health in an individual herd). There is a CellCheck Implementation Group, to facilitate industry engagement and ownership (53). Improvement in national milk quality as measured by BMSCC was observed initially but has subsequently plateaued (Figure 1). In 2020, 65% of herds in Ireland had an annual unadjusted geometric mean SCC <200,000 cells/mL compared with 39% of herds in 2013 (54).

#### Antimicrobial Usage

##### Collection of Antimicrobial Usage Data

The collection of reliable antimicrobial usage data is crucial both for the establishment of antimicrobial stewardship programmes and also as a means to measure their effectiveness (55). In a number of EU member states, substantial progress has been made toward the capture of these data, which has facilitated progress toward national antimicrobial reduction goals. With respect to cattle, there has been full sector coverage in Sweden from 1971 (the Swedish Board of Agriculture (SBA) database), in Denmark from 2000 (VetStat; further detail about VetStat is presented in Table 1), in the Netherlands from 2012 (the “SDa” database; further detail about SDa is presented in Table 1), in Germany from 2014 (the “HIT” database), in Austria from 2015 (PHAROS), and in Belgium from 2017 (the “Sanitel-Med” system) (55, 57). A number of countries publish annual reports of national on-farm antimicrobial usage, including Belgium (58), Denmark (59) and the Netherlands (56). In those countries where national usage data are available, these data are used for multiple purposes including benchmarking of farms and veterinarians and monitoring national and industry-level trends (60).

**Table 1:** Comparison of antimicrobial stewardship in farm animal production in Denmark, Ireland and the Netherlands.

	**Date of introduction**		
	**Denmark^**a**^**	**Ireland^**b**^**	**Netherlands^**c**^**
Ban on prophylactic (preventive) use of antimicrobials	1995	2022^d^	2012
Decoupling of antimicrobial sales and profits	1995		-
Initial restrictions on the on-farm use of antimicrobial agents linked with veterinary oversight, including prescribed farm visits	1995	-	2014
Introduction of requirement for a one-to-one relationship between the farmer and the veterinarian	1995	-	2012^e^
The first annual report of on-farm antimicrobial usage	1996	-	2012
The first treatment/prescribing guidelines to support veterinary clinical decision-making	1996	2022	2012
Mandatory reporting of farm prescribing/usage data to national database	2000^f^	2022^d^	2012^g^
The first restrictions (by industry or government) on the use of highest priority critically important antimicrobials (HP-CIAs) in food animals	2003	2018	2012
The first national target on reduction in antimicrobial usage	2010	–	2009
The introduction of farm-level benchmarking	2010	–	2012
Differential taxes on the sales of antimicrobials and other medicines for veterinary use	2013	–	-
The introduction of prescriber benchmarking	–	–	2012

*The date of introduction of key national measures is presented.*

*^a^Data from DANMAP annual reports. https://www.danmap.org/reports. DANMAP is the Danish Programme for surveillance of antimicrobial consumption and resistance in bacteria from food animals, food and humans*.

*^b^Information from iNAP, Ireland's National Action Plan on Antimicrobial Resistance 2017–2020. https://www.gov.ie/en/publication/ec1fdf-irelands-national-action-plan-on-antimicrobial-resistance-2017-2020*.

*^c^Data from Autoriteit Diergeneesmiddelen (Netherlands Veterinary Medicines Institute, SDa) annual reports. https://www.autoriteitdiergeneesmiddelen.nl/en/publications/general-reports*

*Voluntary from 1995, mandatory for larger farms from 2010.*

*^d^As required under the new Veterinary Medicine Regulation.*

*^e^Introduced in private Integral Chain Control (IKB) systems in 2009, imposed for all farmers by the Product Boards for Livestock, Meat and Eggs (PVE, a public–private organization with legislative powers for the whole livestock sector) in 2012 (56).*

*^f^VetStat was established in 2000, reporting of veterinary prescribing data was mandatory from 2001*.

*^g^Mandatory with cattle from 2012. Implemented in veal calves, broilers and pigs from 2011 (56)*.

In Ireland, national usage data are currently not available. However, this will change with the anticipated introduction of a national electronic prescribing database, as required by the Regulation.

##### Antimicrobial Usage

In Denmark and the Netherlands, there has been substantial progress in reducing on-farm antimicrobial usage in food animal production. In Denmark, the cattle industry set a target to reduce antimicrobial usage in their sector by 20% between 2012 and 2018. In 2017, there was a renewed industry strategy for a 20% reduction in the use of antimicrobials for treatment of mastitis and other cattle diseases as well as lowering geometric mean BMSCC to 150,000 cells/mL by the year 2020. In addition, the dairy industry promoted the use of simple penicillins for DCT and mastitis treatment (61). Overall, there has been a consistently decreasing trend in overall antimicrobial usage in farm animal production in Denmark since 2013, with usage now at its lowest level since 2002 (59). In the Netherlands, national targets were adjusted on several occasions following rapid falls in overall usage of on-farm antimicrobials: by 20% by 2011, by 50% by 2013, and by 70% by 2015, each in comparison to 2009 (62, 63). A 69.9% reduction in overall usage of on-farm antimicrobials in the Netherlands was measured between 2009 and 2019 (64).

Based on sales data compiled by the European Medicines Agency, an estimated 0.2, 0.5 and 1.6 mg/ population correction unit (PCU) of intramammary antimicrobials were used in Denmark, the Netherlands and Ireland during 2018 (65). In Denmark, where DCT is only permitted following confirmation of the presence of mastitis-causing bacteria, just over 30% of cows were treated at drying off in 2019 (59). In Ireland, the equivalent figure was 95%, based on the defined course dose (DCD)/cow per year calculated from national sales data (66). In 2018, the geometric mean BMSCC in Denmark and Ireland were approximately 200,000 (67) and 183,000 cells/mL, respectively (65).

Insights on usage in Ireland are currently derived from national sales data. From a national perspective and based on the sales of veterinary antimicrobials for all livestock species, there was substantial variation in overall usage during 2011–18, from a high of 55.9 mg/PCU in 2013 to a low of 46.0 mg/PCU in 2018 (68). In comparison, the sales of veterinary antimicrobials across all EU member states dropped by more than 35% during this period (68). Intramammary antimicrobial usage in the Irish dairy industry, all based on sales data, is available for the overlapping periods of 2003–10 (54), 2003–15 (69) and 2003–19 (66), and a further 2020 update is currently in press. The estimated on-farm usage of in-lactation therapy has fallen from 0.48 to 0.43 defined course dose (DCDvet, a technical unit of measurement that is commonly used to quantify antimicrobial usage) per cow per year from 2015 to 2019, while DCT has fallen from 1.09 to 0.95 DCDvet per cow per year from 2015 to 2019 (66). In other words, an estimated 95% of Irish cows received DCT at drying off in 2019. In-lactation antimicrobial usage compares favorably with competitor countries, including the UK where reported usage is 0.59 DCDvet per cow per year (70). In contrast, reported national dry-cow antimicrobial usage in the UK, including 0.55 (70) and 0.68 DCDvet per cow per year (71), is considerably lower than in Ireland. In Ireland, almost all in-lactation therapies and approximately 40% of dry-cow therapies contain critically important antimicrobials (CIAs) (19) and there has been an increase in usage of highest priority CIAs [HP-CIAs, essentially equivalent to EMA category B *(“Restrict”)* antimicrobials] in dry-cow therapies in recent years (66).

#### Antimicrobial Stewardship

A range of measures have been introduced in Denmark and the Netherlands to address antimicrobial usage and improve antimicrobial stewardship in food animal production. As highlighted in Table 1 (with further detail in the Supplementary Material), the first of the listed measures was introduced in 1995 in Denmark and in 2012 in the Netherlands. Denmark introduced legislation in 1995 to “decouple” antimicrobial prescribing and sales, limiting veterinary profits from direct sales to 5–10% (72). In the same year, veterinary advisory service contracts were introduced on a voluntary basis, and subsequently mandated in 2010 for larger herds. This requires frequent veterinary visits and a 1-to-1 relationship between the farmer and the veterinarian. Further, farmer access to antimicrobials is linked to the level of farm oversight that is provided by the veterinarian (61). Treatment and control measures are underpinned by an understanding of the aetiologic agent (i.e., bacterial culture), patterns of udder infections and antimicrobial resistance in each herd, and the use of narrow-spectrum antimicrobials and selective DCT has become the norm. This approach is central to basic veterinary training in Denmark (and other Nordic countries), and the same message is conveyed to dairy farmers (67). Farm-level benchmarking has facilitated the introduction of the yellow card system, first in the pig industry, identifying those farmers, for remedial actions, with the highest consumption of antimicrobials per animal produced (73). In the Netherlands, independent evaluation of the concept of decoupling was conducted in 2010. It was concluded that economic motives to prescribe would best be addressed by introducing a strict 1-to-1 farmer-veterinarian relationship (in place from 2010) and prescriber benchmarking (from 2013) (62). Since 2012, there has been a mandatory requirement to register all antimicrobials supplied by veterinarians (74), and benchmarking, both of farmers and prescribers, has been identified as a critical factor for success in prudent prescribing. Benchmarking is generally viewed positively by Dutch veterinarians (75). In 2012, prophylactic use of antimicrobials was banned in the Netherlands, and the use of HP-CIAs (3rd and 4th generation cephalosporins, fluoroquinolones) in farm animals was prohibited, apart from exceptional circumstances (62). Since 2012, SDa (Autoriteit Diergeneesmiddelen, Netherlands Veterinary Medicines Institute) have produced an annual report of antimicrobial usage in agricultural livestock (56). Guidelines for the implementation of selective DCT were introduced in 2014 (76).

In Denmark, measures to reduce antimicrobial use were mainly established and carried out by the government, following consumer concerns about antimicrobial use (62). A strong level of trust among farmers, consumers, educators, researchers and governmental agencies has enabled strict legislation and recommendations on antimicrobial use in food animal production to be introduced and implemented. This approach has received strong societal support, with progress reliant on the willingness of all stakeholders to cooperate (67). In the Netherlands, multiple events contributed to national change, including consumer concern following the discovery of a reservoir of multidrug resistant *S. aureus* (MRSA) in the livestock sector (77). The Dutch government acted as a facilitator for change, coupled with self-regulation by private parties. In the Netherlands, the government set national reduction targets, co-funded SDa, incorporated private regulations into legislation, intensified inspection and enforcement of legislation and supported the strengthening of the independent position of veterinarians through the introduction of technical measures of antimicrobial prescribing and usage (62). For comparison, in New Zealand, where selective DCT is now widely practiced, change was driven solely by industry, without any regulatory oversight (78).

In Ireland during 2018, there was a cessation in the sale of intramammary products containing HP-CIAs from many points of retail and the development of a national policy on the use of HP-CIAs in food animals (46). Further, prescribing guidelines for private veterinary practitioners (PVPs) were introduced in 2022 (49) (Table 1).

### International Scientific Reviews

A broad range of literature relevant to mastitis control and intramammary antimicrobial stewardship was considered, including material cited. In addition, the recent RONAFA opinion (79) provides a detailed overview of measures that have been implemented across EU member states to reduce the need to use antimicrobial agents in animal husbandry. Measures of particular relevance to Ireland are also available (60).

### Additional Concepts

#### Bulk Milk Somatic Cell Counts as an Indicator of Intramammary Infection

BMSCCs provide a general indication of the level of intramammary infection (IMI) in dairy herds. It is a crude measure of cow-composite SCCs, and therefore is a crude measure of the prevalence of IMI within a herd (80). A farm with BMSCC consistently above 200,000 cells/mL (the industry's most common threshold) or either a sharp or gradual increase can signal the existence of a mastitis problem (81), especially in herds with a contagious mastitis problem (82).

Multiple factors influence the association between BMSCC estimates and within-herd IMI prevalence, including the udder pathogen(s) involved (for example, *Streptococcus agalactiae* will more strongly stimulate a cellular reaction than *Staphylococcus aureus*), the strictness with which milk from cows with clinical mastitis is kept out of the bulk tank, the age of the cows, the stage of lactation and the average herd production level (83). Although care is needed with interpretation, in the context of udder pathogens such as *S. aureus*, higher annual BMSCC estimates are generally indicative of herds with suboptimal mastitis control.

BMSCC thresholds vary between countries. In a meta-analysis investigating the efficacy of selective and blanket DCT, the most common herd-level selection criteria was to have a BMSCC below a predetermined threshold (ranging from 250,000 to 400,000 cells/ml) (84). Ruegg (81) suggested that consistent production of milk with BMSCC values <200,000 cells/mL is an achievable goal for most dairy farms.

#### Detecting Intramammary Infection in the Individual Cow

Many tests have been evaluated for the diagnosis of subclinical mastitis, which is defined as the presence of inflammation with a normal appearance of the mammary gland and visibly normal milk (85). Reflecting the inflammatory status of the mammary gland, milk SCC is used extensively to monitor udder health and milk quality (85, 86). It is associated with the risk of IMI, both at the level of the quarter and the cow (87), and a cow-composite SCC of >200,000 cells/mL is a strong indicator of mastitis (81). As reported by McDougal (87), cow-composite SCC has a diagnostic sensitivity (Se) of 0.3–0.85 and specificity (Sp) of 0.67–0.9 of predicting IMI, given a cut point of 200,000 cells/mL. The California mastitis test (CMT) is commonly used to diagnose IMI (17). However, if the CMT were used to aid selection of cows suitable for selective DCT, a large percentage of uninfected quarters would receive DCT (23–46% of cows without infection with major udder pathogens were detected by CMT) (88, 89). Culture (at the laboratory and on-farm) and real-time multiplex polymerase chain reaction (PCR) can be useful in the diagnosis of an IMI. On-farm culture systems generally seek to categorise udder pathogens into broad categories, as an aid to treatment rather than a means to make species-level pathogen diagnoses (85). Compared with culture, PCR is both faster and more sensitive, but more costly and with the potential to detect DNA from dead bacteria (85). Machine learning algorithms, to aid analysis of the large amounts of farm data that are generated, offer a promising new tool to support farm-level decision-making (86), including predictive algorithms for intramammary infection status in late-lactation cows (90).

#### Mastitis Control and Intramammary Antimicrobial Stewardship

Mastitis is often associated with low levels of hygiene during milking (e.g., wearing of gloves for milking, milking clean teats and appropriate post milking teat disinfection) or at dry-off (3, 91, 92) as well as with general farm management practices (93). In comparison to higher BMSCC herds, lower BMSCC herds are more likely to implement management practices that are conducive to improved mastitis control (including attention to hygiene; cleaner cubicles, drinking buckets and cows; cleaner calving pens and drier bedding for lactating cows in maternity pens; post-milking disinfection; DCT) (94). In the case of contagious mastitis, ongoing infection pressure will facilitate within-herd transmission, potentially placing younger, non-infected cows at risk, even with careful attention to detail during milking. This discussion is particularly important in the context of the Regulation, which states that antimicrobials “*shall not be applied routinely nor used to compensate for poor hygiene, inadequate animal husbandry or lack of care or to compensate for poor farm management”* (12). The majority of antimicrobial usage in dairy herds is associated with mastitis treatment and control (95), and optimal mastitis control is central to efforts to reduce on-farm antimicrobial usage.

## Specific Irish Challenges

### Mastitis Control

#### Suboptimal Mastitis Control

Based on the most-recent estimates from 2020, 35% of Irish herds had an annual unadjusted geometric mean BMSCC of 200,000 cells/mL or greater, suggestive of the potential for suboptimal mastitis control in these herds. National progress toward improved mastitis control has slowed in recent years (Figure 1).

#### Predominant Udder Pathogens

*S. aureus, Streptococcus uberis* and *Escherichia coli* are commonly isolated udder pathogens on Irish dairy farms (96, 97). There is a high prevalence of *S. aureus* infection on Irish farms.

#### Milk Recording

The level of milk recording in Ireland (in 2017: 33% of herds and 48% of cows) is low relative to international counterparts (98). Milk recording provides animal-level information to inform both mastitis control and prudent prescribing.

#### Seasonal Dairy Production

Dairy production is highly seasonal on most Irish farms, which adds complexity to herd management at specific periods of the year (99). An estimated 1 million dairy cows are dried off in Ireland over an 8-week period.

#### Labour Shortages

There are ongoing difficulties in sourcing experienced labour (on dairy farms, as milking technicians, for bulk tank servicing), exacerbated by a period of recent expansion in the Irish dairy industry. These challenges can substantially complicate farm management, including those activities contributing to mastitis control and intramammary antimicrobial stewardship.

#### Housing Challenges

In 2017, 32.9% of surveyed Irish dairy farmers provided <1 cubicle per cow throughout the winter housing period (100). Only 2.5% of surveyed farmers had more than 1 cubicle per cow (101). These housing constraints have implications for cow welfare and mastitis control.

#### Limited Scope in Mastitis Investigations

Detailed mastitis investigations are not routinely conducted in response to on-farm mastitis problems, either by veterinary practitioners or other farm advisors. Consequently, on-farm recommendations can be generic in nature, rather than informed by a detailed understanding of the epidemiology of infection or of broader drivers for mastitis on the farm in question. This is changing, in part with the introduction of the Graduate Certificate in Dairy Herd Health for veterinary practitioners at University College Dublin (102).

#### National Regulations

A series of data adjustments and interpretation are applied in Ireland to determine herd eligibility to supply raw milk for processing of dairy products. Legislation is currently being interpreted in a manner that facilitates ongoing supply (103, 104), which may reduce the imperative to sustainably address mastitis issues in herds with suboptimal mastitis control.

#### Private Standards

Most Irish dairy farmers are members of the Bord Bia Sustainable Dairy Assurance Scheme (SDAS), which is a national quality assurance programme that includes a range of private animal health and welfare standards (105, 106). Although SDAS has the potential to motivate farmers toward improved milk quality, this potential has not yet been realised because the SDAS standards do not exceed the legislative baseline with respect to milk quality (106).

#### Objective Assessment of Progress

National BMSCC data is not yet available for public good research. Consequently, it is not possible to answer key questions to help inform national decision-making on progress in national mastitis control, the CellCheck programme or antimicrobial stewardship in Ireland.

### Intramammary Antimicrobial Usage and Stewardship

Each of the issues raised in the previous section (Mastitis control) is relevant, either directly or indirectly, to intramammary antimicrobial usage and stewardship. In addition:

#### Remote Prescribing

In Ireland between 2007 and 2022, remote prescribing of intramammary antimicrobial products was allowed under national legislation (107), leading to the potential for very limited veterinary oversight of these products. Although this legislation is now revoked (108), there may be a legacy impact on the attitudes and behaviours of farmers and others within the dairy industry, with implications for future efforts toward improved intramammary antimicrobial stewardship.

#### The Potential for Multiple Prescribers

In Ireland, prescribing must be undertaken by a veterinary practitioner; this is generally the attending veterinary practitioner (or group of veterinary practitioners) who have been given responsibility for the primary care of a herd by the designated keeper in the context of a client-patient-practice relationship (CPPR) (“*an agreement between an animal owner (or designated keeper) and a veterinary practitioner(s) within a veterinary practice to provide veterinary services that demonstrate real and ongoing clinical veterinary practitioner/animal contact”*) (109). However, a herdowner may have more than one CPPR in place, thereby allowing for the potential for antimicrobials to be sourced from more than one prescriber. In addition, between 2007 and 2022, remote prescribing of intramammary antimicrobial products was allowed.

#### Limited Insights Into On-Farm Usage

Ireland is currently reliant on national sales data to estimate on-farm intramammary antimicrobial usage. Given the nature of these data, farm-level usage cannot be calculated, and prescriber and farm-level benchmarking are not currently possible.

#### Historic Absence of Selective DCT

Based on ongoing analyses of national sales data, there has been minimal shift to this point from blanket to selective DCT on Irish dairy farms (66).

#### Increasing Use of EMA Category B (“Restrict”) Antimicrobials

There is evidence of ongoing usage of EMA category B *(“Restrict”)* antimicrobials in in-lactation therapy and of increasing usage of these antimicrobials in DCT (66).

## TWG Recommendations

The following section outlines the pragmatic national and farm-level recommendations that were developed by the CellCheck TWG in support of improved mastitis control and intramammary antimicrobial stewardship in Ireland. These recommendations are cognisant of and informed by all of the preceding information presented in this paper, and many of these recommendations are in place in other dairying countries.

These recommendations were initially presented to the Veterinary Council of Ireland (VCI), the national regulator of the veterinary profession in Ireland. This is in response to a request in early 2021 from the VCI for technical perspectives on the application of the Regulation in Ireland. This VCI submission is available as Supplementary Material.

### National Actions

It is recognised that a comprehensive range of national actions are required to support optimal mastitis control and antimicrobial stewardship across all Irish farms.

#### A Review of Regulatory and Non-regulatory Drivers of On-Farm Mastitis Control

- The regulatory drivers for change need to be reviewed. The criteria for herd eligibility to supply needs to be redrafted, including the corrective action required and the performance to be achieved when milk quality standards are not met, to require all farms to sustainably resolve milk quality issues.
- There is a need to leverage Bord Bia SDAS standards to facilitate improved milk quality.
- Detailed supporting research is needed to inform national decision-making on progress in national mastitis control, the CellCheck programme and antimicrobial stewardship in Ireland. To date, such analyses have not been possible, as the relevant BMSCC data are not available for public good research.

#### Monitoring and Restricting Antimicrobial Usage

- Detailed monitoring of on-farm antimicrobial usage is needed, including objective measurement, systems for benchmarking that are understandable to the user, and defined thresholds for further investigation. This is needed at multiple levels: nationally, at the level of the prescribing veterinary practice, and at farm-level. Electronic capture of prescribing data will be central to these efforts.
- Restrictions and/or bans on the use of specific antimicrobials within the dairy industry are needed, specifically EMA category B *(“Restrict”)* antimicrobials.

#### Professional Oversight

- On farms under their care, prescribers need knowledge and professional oversight of all of the antimicrobials that are prescribed and used. The practical approaches used to achieve this should be consistent with international best-practice in antimicrobial stewardship.
- Ongoing education and training for prescribers is needed, to maximise their knowledge and impact across a range of areas, including detailed mastitis investigations, CellCheck resources and tools and prudent antimicrobial stewardship.
- Drawing on European best-practice, there is a need for detailed treatment guidelines for veterinarians to help guide the responsible use of antimicrobials in different clinical scenarios.
- A national discussion is needed of the multiple potential conflicting interests that veterinarians face when making prescribing decisions, including the professional obligations to alleviate suffering while ensuring prudent prescribing, but in the context of the prescriber's financial dependency on clients from the sale of antimicrobials and of the need for risk avoidance.

#### Supporting Infrastructure

- There is a need to ensure a network of laboratories with sufficient capacity and expertise to deliver bacterial culture and antimicrobial sensitivity testing, and for these data to be available to enable integrated reporting.
- There is a need to leverage ongoing advances in data management and analysis, using milk quality data (BMSCC, milk recording and milk culture results), to facilitate the development and improvement of tools to assist with national and farm-level decision-making.
- There is a need for ongoing support for farm-level mastitis control, through farmer education [conducted as a collaborative effort by PVPs, Teagasc (the Agriculture and Food Development Authority), milk processors and the national Department of Agriculture, Food and the Marine (DAFM)], farmer peer learning through discussion groups, supportive milk pricing structures, and technological developments (including farm-level dashboards and economic cost calculators).

#### Additional Actions

- There must be clear, meaningful industry-agreed targets that reflect both the ambition of the industry and the changes that need to happen to maximise udder health while reducing the use of antimicrobials in compliance with the Regulation.
- A comprehensive industry plan is needed to increase awareness and education of responsible antimicrobial use and future legislative change, along with practical supports and resources to enable farmers to make the necessary transition.
- National efforts are needed to facilitate a team-based approach to milk quality, with prescribers working collaboratively with other professional farm service providers, including farm advisors and milking machine technicians.

### On-Farm Actions

#### At Drying off

Under the Regulation, prophylactic use must not be routinely carried out. Further, “*antimicrobials should not be applied routinely nor used to compensate for poor hygiene, inadequate animal husbandry or lack of care or to compensate for poor farm management”* (12). In compliance with this Regulation, DCT should be limited to therapeutic use in cows known to be infected. Further, the EMA guidelines should be followed, providing an overview of the categorization of antimicrobials for use in animals for prudent and responsible use (18).

The use of selective DCT may not increase the risk of intramammary infection at calving if internal teat sealants are used for all cows (110). As highlighted previously, high standards in hygiene will be critical during the administration of teat sealant (23), and throughout the dry period and around calving. Although infection risks relating to the milking parlor are removed during the dry period, higher BMSCC herds and those with high incidence of infections may also have suboptimal management conditions which may be associated with an increase in infectious challenge during the dry period and around calving.

There is a need for individual cow information to determine infection status at drying off. Currently, milk recording is recommended every 4–6 weeks in Ireland, with a minimum of 6 recordings throughout the lactation including one shortly prior to drying off and one shortly following calving. At this frequency, data are sufficient to support both mastitis control and prudent prescribing. The TWG recognise that other options are also possible (such as bacterial culture, PCR testing, California mastitis testing) (80, 111, 112), but with disadvantages. Further, studies from the Netherlands (113) and New Zealand (87) suggest that a single milk recording, taken within 4–6 weeks of drying off, can provide useful information about infection status at drying off. However, this limited information would be insufficient to guide mastitis control on Irish herds throughout the year.

There is also a need for herd-level information, including knowledge and oversight by the prescriber of all antimicrobials prescribed and used, and of the rationale and strategy for antimicrobial prescribing and use. This is best achieved, consistent with international best-practice in antimicrobial stewardship, with a single prescriber (or single prescribing practice) for each farm. There is also a need for a detailed understanding by the prescriber of the farm, including the herd, the people, the facilities, and farm management (in general, during lactation and at drying off). This information is central to mastitis management, including the investigation of suboptimal mastitis control, to gain an improved understanding of the epidemiology of infection and of factors contributing to suboptimal mastitis control based on a detailed on- and off-farm investigation, to develop and agree a plan with the farmer to robustly and sustainably address each of these factors, including agreed actions and timelines and objective measures to monitor progress, and for ongoing and regular assessment and review. It is important that the prescriber has an understanding of milk quality trends, the mastitis pathogen challenge and antimicrobial sensitivity/resistance patterns, to inform prescribing.

Guidelines for prescribers to support prescribing and mastitis control decisions at drying off are presented in Table 2. In addition, see (49).

**Table 2:** Proposed guidelines for the prescribing private veterinary practitioner (PVP) to support prescribing and mastitis control decisions at drying off.

**Lower risk herds** ***(Those where there is objective evidence that mastitis is under good control and the prevalence of infection is consistently low)***		
	**Milk recording**	**No milk recording**
Prescribing decisions	Follow the current CellCheck Dry Cow Strategy^a^. Make prescribing decisions informed by: • Individual animal information *(as outlined in the text)*, • Herd-level information *(as outlined in the text)*, and • European Medicines Agency (EMA) guidelines.	In the absence of milk recording data, the prescribing PVP should use the following to identify individual cows that have evidence of infection, and therefore require antimicrobial treatment: • A single milk recoding from each cow within 4–6 weeks of drying off, or • Individual milk culture results, or • Individual California Mastitis Test (CMT), as carried out by the prescriber. Prescribing decisions should be made using this information, informed by: • The current CellCheck Dry Cow Strategy, • Herd-level information *(as outlined in the text)*, and • EMA guidelines. A comprehensive whole herd milk recording programme should commence with the start of the next lactation.
Mastitis control decisions	Provide professional support to maintain optimal mastitis control. At the time of dry-cow prescribing: • conduct a review of treatment of in-lactation cases in the past season and • develop/agree a standard operating procedure for the treatment of in-lactation cases in the following season.	The farmer should immediately commence comprehensive whole herd milk recording. Provide professional support to maintain optimal mastitis control. At the time of dry-cow prescribing: • conduct a review of treatment of in-lactation cases in the past season, and • develop/agree a standard operating procedure for the treatment of in-lactation cases in the following season.
**Higher risk herds (** ***All other herds*** **)**		
Prescribing decisions	Follow the current CellCheck Dry Cow Strategy, with consideration to reduce the individual cow SCC threshold for antimicrobial treatment. Make prescribing decisions informed by: • Individual animal information *(as outlined in the text)*, • Herd-level information *(as outlined in the text)*, and • EMA guidelines. • If the prescribing PVP may consider that prophylactic use of dry-cow antimicrobial is justified in order to protect cow welfare, in situations where the risk of new infection over the dry period is unacceptable, it is critical that these risk factors are addressed and resolved, certainly prior to the next dry period.	In the absence of milk recording data, the prescribing PVP should use the following to identify individual cows that have evidence of infection, and therefore require antimicrobial treatment: • A single milk recoding from each cow within 4–6 weeks of drying off, or • Individual milk culture results, or • Individual CMT, as carried out by the prescriber. Prescribing decisions should be made using this information, informed by: • The current CellCheck Dry Cow Strategy, • Herd-level information *(as outlined in the text)*, and • EMA guidelines. If the prescribing PVP may consider that prophylactic use of dry-cow antimicrobial is justified in order to protect cow welfare, in situations where the risk of new infection over the dry period is unacceptable, it is critical that these risk factors are addressed and resolved, certainly prior to the next dry period. A comprehensive whole herd milk recording programme should commence with the start of the next lactation.
Mastitis control decisions	The farmer should engage with their PVP and other milk quality professionals to sustainably resolve constraints to effective mastitis control. Each of the following will be needed: • A detailed understanding of the epidemiology of infection and of factors [including cause(s) and driver(s)] contributing to suboptimal mastitis control based on a detailed on- and off-farm investigation, • A plan developed and agreed with the farmer to robustly and sustainably address each of these factors, including agreed actions and timelines and objective measures to monitor progress, and • Ongoing and regular assessment and review. At the time of dry-cow prescribing: • conduct a review of treatment of in-lactation cases in the past season, and • develop/agree a standard operating procedure for the treatment of in-lactation cases in the following season.	The farmer should immediately commence comprehensive whole herd milk recording. The farmer should engage with their PVP and other milk quality professionals to sustainably resolve constraints to effective mastitis control. Each of the following will be needed: • A detailed understanding of the epidemiology of infection and of factors [including cause(s) and driver(s)] contributing to suboptimal mastitis control based on a detailed on- and off-farm investigation, A plan developed and agreed with the farmer to robustly and sustainably address each of these factors, including agreed actions and timelines and objective measures to monitor progress, and • Ongoing and regular assessment and review. At the time of dry-cow prescribing: • conduct a review of treatment of in-lactation cases in the past season, and • develop/agree a standard operating procedure for the treatment of in-lactation cases in the following season.

a *https://animalhealthireland.ie/assets/uploads/2021/04/CellCheck-Dry-Cow-Strategy-July-2019.pdf*.

#### During Lactation

Animal- and herd-level information will again be required. The TWG recommends ongoing collection and testing of milk samples from animals with clinical or subclinical mastitis, ensuring a mixture of young and old cows, with evidence of both recent and chronic infections, based on sampling conducted at different points throughout lactation, to identify the causative pathogen(s). This is to guide individual clinical decisions and, equally importantly, as part of the broader assessment and monitoring of mastitis pathogen challenge(s) and antimicrobial resistance patterns on the farm.

The CellCheck TWG recommendations to support prescribing and mastitis control decisions during lactation are presented in Table 3. In addition, see (49).

**Table 3:** Proposed guidelines for the prescribing private veterinary practitioner (PVP) to support prescribing and mastitis control decisions during lactation.

	**Farm mastitis pathogen challenge(s)/antimicrobial resistance patterns are known**	**Farm mastitis pathogen challenge(s)/antimicrobial resistance patterns are not known**
Prescribing decisions	Confirm diagnosis of mastitis during lactation, by clinical examination or other proper assessment Select appropriate antimicrobial, based on both cow and farm factors: • Cow factors such as clinical findings, lactation number and treatment history, and • Farm factors such as farm udder pathogen profile, antimicrobial susceptibility testing (AST) and previous treatment outcomes. Choose an antimicrobial from the lowest category possible on the EMA Antimicrobial Advice Ad Hoc Expert Group (AMEG) list that has been shown to be effective, given knowledge of the farm mastitis pathogen challenge(s) and antimicrobial resistance patterns.	Confirm diagnosis of mastitis during lactation, by clinical examination or other proper assessment. Choose an antimicrobial from the ‘*EMA Category D: Prudence'* category. Antimicrobials from ‘higher' categories *(EMA Categories B: Restrict, C: Caution)* should only be considered with supporting milk culture and antimicrobial susceptibility results and only when there are no antimicrobials in a lower category that could be clinically effective.
Mastitis control decisions	Mastitis events, treatments administered, and related outcomes should be recorded by the farmer and made available to the PVP for analysis to assist with future treatment decisions. In-lactation mastitis incidence should be monitored. Develop/conduct an annual review of a mastitis treatment plan for in-lactation cases.	Mastitis events, treatments administered, and the related outcomes should be recorded by the farmer and made available to the PVP for analysis to assist with future treatment decisions. In-lactation mastitis incidence should be monitored. Instigate measures to gain a detailed knowledge of the udder pathogen challenge(s) and antimicrobial resistance patterns on the farm. This should include ongoing collection and analysis of the following milk samples, which may be frozen if necessary: • A pre-treatment milk sample from all clinical cases, and • Milk samples from cows with high individual SCC, ensuring a mixture of young and old cows, with evidence of both recent and chronic infections. Develop/conduct an annual review of a mastitis treatment plan for in-lactation cases.

## Discussion

This paper provides an overview of the issues that were considered by the CellCheck TWG during the development of pragmatic national and farm-level recommendations for improved mastitis control and intramammary antimicrobial stewardship in Ireland. The issues were broad-ranging, including consideration of policy drivers, comparison with international best-practice, international scientific reviews and specific Irish challenges. This work was undertaken in anticipation of the Veterinary Medicine Regulation, in force from 28 January 2022. The Regulation has generated considerable political interest in Ireland, including a recent report further to a series of submissions to the Oireachtas Joint Committee on Agriculture and the Marine (114).

In recent years, substantial progress has been made in support of efforts toward improved mastitis control and antimicrobial stewardship in the Irish dairy industry. As highlighted in Figure 1, there has been a substantial improvement in milk quality, with 65% of herds with an annual geometric mean BMSCC below 200,000 cells/mL. The CellCheck programme has been working to assist industry to change behaviour in relation to antimicrobial usage. Based on national sales data, there has been a substantial improvement in national udder health and a concurrent reduction in in-lactation AM usage (66). Further, since autumn 2018, CellCheck Dry Cow Consults have been introduced, providing an opportunity for farmers and their trained veterinary practitioner to review lactation and dry period performance data, the drying off process and dry cow management, and to identify cows that could be dried off without antimicrobial treatment (115). These consults have been delivered as part of the Targeted Advisory Services in Animal Health (TASAH), with funding provided by the Rural Development Plan 2014–2020. In addition, in partnership with industry stakeholders, there has been much CellCheck activity including regular communications, on-farm events and service provider training to build the awareness, knowledge and capacity around reducing antimicrobial use, particularly at drying off.

The case studies from Denmark and the Netherlands have been of assistance, highlighting pragmatic national and on-farm approaches that have been used in support of prudent prescribing practices and meaningful reductions in antimicrobial usage. There are similarities between the dairy industries in these countries and in Ireland, which highlights the relevance of these comparisons. In Denmark and the Netherlands, there has been a shared objective and an agreed, proactive and integrated national approach that has been rolled out over a series of years. Key actions, including those shared in both countries, include a ban on the prophylactic use of antimicrobials, a national database of antimicrobial usage allowing objective measurement and benchmarking and transparent reporting (nationally, by sector, on each farm and with each prescriber), clarity on the level of veterinary oversight required (such as mandatory veterinary visits, one-to-one relationships, annual evaluation of farm health and treatment plans), detailed treatment guidelines, national reduction targets in antimicrobial usage, and restrictions on the usage of specific antimicrobials.

In this paper, we propose a series of national and on-farm recommendations to support mastitis control and intramammary antimicrobial stewardship in Ireland. In many key areas of concern, the TWG recognises the challenges in seeking to shape recommendations in the absence of robust and practical scientific evidence. For this reason, some of the recommended actions are pragmatic in nature, informed by national and international experiences. Periodic programme review will be needed, informed by ongoing monitoring of key performance indicators, to identify those actions that are most effective in an Irish context.

## Author Contributions

The concepts in this paper were conceived during detailed discussions within the CellCheck TWG, of whom all coauthors are members. SM wrote the paper, with detailed contributions from CM, PSB, LO'G, and FM. All authors reviewed and approved the final manuscript.

## Author Disclaimer

The views, information and opinions expressed in this paper are solely those of the co-authors and do not necessarily represent those of Animal Health Ireland or the co-authors' organisations of employment.

## Conflict of Interest

The authors declare that the research was conducted in the absence of any commercial or financial relationships that could be construed as a potential conflict of interest.

## Publisher's Note

All claims expressed in this article are solely those of the authors and do not necessarily represent those of their affiliated organizations, or those of the publisher, the editors and the reviewers. Any product that may be evaluated in this article, or claim that may be made by its manufacturer, is not guaranteed or endorsed by the publisher.

## Supplementary Material

The full CellCheck TWG proposal to support intramammary antimicrobial stewardship in Ireland, as submitted to the Veterinary Council of Ireland, is available as Supplementary Material.
