# Supplementary material for: Mastitis Control and Intramammary Antimicrobial Stewardship in Ireland: Challenges and Opportunities
Source: Front Vet Sci. 2022 Apr 11;9:748353. doi: 10.3389/fvets.2022.748353 (PMC9040554; doi:10.3389/fvets.2022.748353)
Supplement: Supplementary file 1 [file Data_Sheet_1.docx]

**Guidance document for PVPs in prescribing dry-cow and in-lactation antibiotics**

*CellCheck technical working group, 27 April 2021*

**Purpose**

In response to our engagement with the Veterinary Council of Ireland, the CellCheck technical working group (TWG) was asked to provide detailed science-based prescribing guidelines for private veterinary practitioners (PVPs) in the context of the new Veterinary Medicine Regulation. This guidance document has been informed by international (and particularly European) best-practice (see Appendix 1; EMA/EFSA, 2017; More, 2020), by international and Irish research (including Devitt et al., 2013; More et al., 2013, 2017; McAloon et al., 2021), and by the collective knowledge and experience of Animal Health Ireland’s CellCheck technical working group.

Therefore, the purpose of this document is to provide guidance for PVPs when prescribing:

- Dry-cow intramammary antibiotics for herds with and without milk recording data, and
- In-lactation antibiotics for herds with and without information on farm-level mastitis pathogen challenge(s)/antibiotic resistance patterns.

It also outlines the comprehensive range of national actions that will be required to support optimal mastitis control and responsible prescribing across all Irish farms.

**Introduction**

The udder health of the national herd has improved significantly over the last decade, putting the industry in a stronger position as it prepares to implement new Veterinary Medicine Regulations. This national improvement in mastitis control and milk quality has been based on key principles of creating awareness, establishing best practice, building capacity and setting goals, along with industry collaboration. Established in late 2010, CellCheck has played a critical role in facilitating the industry to work together towards this common goal. Such progress would not be possible without the contribution of bodies representing farmers, processors, service providers and government, and partnerships at farm level of farmers, co-op and farm advisors, milking machine technicians and vets.

The new Veterinary Medicines Regulation (2019/6) presents challenges in all Irish dairy herds, but particularly in herds where mastitis control is suboptimal. In comparison to herds where mastitis control is optimal, there are more infected cows in higher somatic cell count (SCC) herds and a greater need for therapeutic usage of antibiotics, both in-lactation and at drying-off. In these herds, a reduction in intramammary antibiotic usage (both in-lactation and at drying-off) will lead to increased risks for cow welfare. Further, a shift away from prophylactic antibiotic usage at drying-off (as required under the new Veterinary Medicines regulation) will make herd-level control more challenging by reducing the opportunities for cure and prevention over the dry period, although the latter can be mitigated with teat sealants when applied correctly. For cows classified as non-infected within these herds, such a shift would also be associated with greater inherent risk for these animals, given the higher uncertainty of their infection status when treatment decisions are being made, often combined with sub-optimal management conditions and increased infectious challenge during the dry period.

In these higher SCC herds, there are challenges in seeking a balance between ensuring legislative compliance and managing welfare risk. Indeed, there is a need for balance between three differing objectives:

- An ongoing need for therapeutic treatment of infected cows, both in-lactation and at drying-off.
- The requirement under the new Veterinary Medicines regulation to cease any further antibiotic use as a prophylactic measure. Antibiotic usage must be justified on therapeutic grounds, with prophylaxis only allowed under exceptional circumstances.
- An overriding need to improve herd-level mastitis control to reduce on-farm antibiotic usage.

For all herds, there is also the risk that in an effort to reduce antimicrobial use on farm, farmers may seek to use alternative products, with little or no evidence to support their efficacy. It is crucial that treatment and management decisions are supported by independent science.

In the Animal Health Ireland (AHI) CellCheck Dry Cow Strategy (see Appendix 2), recommendations for selective dry-cow therapy (sDCT) are currently proposed for herds where there is evidence of good mastitis control, for example, unadjusted bulk tank SCC is consistently ≤200,000 cells/mL. In higher SCC herds, some of the infection risk associated with sDCT may be mitigated by lowering the recommended individual cow treatment threshold, for example, from 100,000 to 50,000 cells/mL. For these latter herds, there is an imperative for meaningful engagement with PVPs and other professionals to sustainably lower the overall mastitis risk for the herd. Otherwise, the ability to safely reduce the level of antibiotic use at drying off and in-lactation will be limited. This engagement will need to at least consider each of the following:

- A detailed understanding of the factors (including cause(s) and driver(s)) contributing to suboptimal mastitis control based on a detailed on- and off-farm investigation,
- A plan developed and agreed with the farmer to robustly and sustainably address each of these factors, including agreed actions and timelines and objective measures to monitor progress,
- Ongoing and regular assessment and review.

The ongoing problem of sub-optimal mastitis control in the national herd must be resolved as a matter of urgency. This would substantially and sustainably resolve each of the challenges posed by the new Veterinary Medicines regulations, in addition to multiple other benefits. However, the challenge is substantial, with sub-optimal mastitis control likely on an estimated 33% of Irish herds (30% of herds with annual geometric mean unadjusted SCC >200,000 cells/mL; 3-4% of herds >400,000 cells/mL; collectively approximately 5,000 herds). The current document is underpinned by the fundamental need for national action to drive change towards improved farm-level mastitis control, including the provision of suitable information to support risk-based decision making and a network of support to improve/drive improved on farm activities such as hygiene. This is outlined in detail below.

Antibiotics can be used in three ways:

- Therapeutic use, which refers to treatment given to animals with evidence of infection.
- Metaphylactic use as defined in 2019/6 means the administration of a medicinal product to a group of animals after a diagnosis of clinical disease [bacterial infection] in part of the group has been established, with the aim of treating the clinically sick [infected] animals and controlling the spread of the disease to animals in close contact and at risk and [treating animals] which may already be subclinically infected. [During metaphylactic use, the true infection status of all treated, non-clinically sick animals is unknown, as distinct from therapeutic use for subclinical infections use or prophylactic use in uninfected animals (see below)]
- Prophylactic use (preventive treatment) as defined in 2019/6 means the administration of a medicinal product to an animal or group of animals before clinical signs of a disease, in order to prevent the occurrence of disease or infection.

Antibiotic treatment during lactation

During lactation, treatment is primarily only administered to known infected animals (either clinical or subclinical) and is therefore deemed therapeutic use. Metaphylactic use is rarely necessary during lactation, only given in response to large-scale outbreaks of highly contagious mastitis. This is a rare event and generally related to sub-optimal hygiene, farm management or husbandry. However, as clearly articulated in Article 107(1) of 2019/6, antibiotics should not be used to compensate for poor hygiene, inadequate animal husbandry or lack of care or to compensate for poor farm management.

Antibiotic treatment at drying off

Based on the definitions above, traditional blanket dry-cow therapy (the routine treatment of all animals with antibiotic therapy) can either be classified as therapeutic or prophylactic usage, depending on the cow’s true infection status. It has been suggested that dry cow therapy can also constitute metaphylactic usage, as the true infection status is not always unknown. However, this term is not appropriate for dry cow therapy because the milking process has ceased and the primary risk factor for the spread of contagious mastitis has been removed.

Under the new Veterinary Medicines Regulation, prophylactic use must not be routinely carried out, which is a substantial departure from current practice for dry-cow therapy in Ireland. Prophylactic dry-cow therapy is primarily used to compensate for sub-optimal hygiene, farm management or husbandry, either during the process of administration of dry-cow therapy or over the dry-period. However, as highlighted above (Article 107(1) of 2019/6), antibiotics should not be applied routinely nor used to compensate for poor hygiene, inadequate animal husbandry or lack of care or to compensate for poor farm management. In the future, the prescribing of dry-cow therapy in compliance with this Regulation will only be acceptable based on therapeutic use in cows known to be infected.

**Animal-level information necessary for responsible prescribing**

1. Individual assessment of specific animals with clinical or subclinical mastitis
   1. Cases of clinical mastitis, often in-lactation, are identified based on clinical signs of inflammation/systemic illness, or abnormal changes in the appearance of milk. Subclinical infection is diagnosed on the basis of individual cow SCC levels. There should also be ongoing collection and testing of milk samples from animals with clinical or subclinical mastitis, both to guide individual clinical decisions and equally importantly as part of the broader assessment and monitoring of mastitis pathogen challenge(s) and antibiotic resistance patterns on the farm, as outlined below.
2. Ongoing assessment of all lactating animals
   1. Milk recording. The TWG recommends milk recording every 4-6 weeks as best practice, for prescribing decisions and mastitis monitoring, with a minimum of 6 recordings throughout the lactation^[[1]](#footnote-1)^, including one shortly prior to drying off and one shortly following calving. Milk recording results are currently accepted internationally as best-practice in identifying cows at the end of their lactation with probable infected quarters, and for prescribing dry-cow antibiotic treatment. While it may be possible to improve the accuracy of diagnosing infection through the additional measures of clinical disease history, bulk tank SCC trends and milk culture results, milk recording is a mainstay of this decision-making process, due to the relative practicality of delivery and multiple additional benefits to farm management^[[2]](#footnote-2)^.
   2. Alternatives to milk recording are available, but are currently of limited practical value:
      1. Bacterial culture or polymerase chain reaction (PCR)-based techniques are the gold standard for indicating presence of bacteria or DNA, respectively, and thus may be proposed as suitable individual cow information for the purposes of prescribing dry-cow antibiotic. However, in Irish farming systems with seasonal calving and hence block drying off, the logistics of aseptically sampling large numbers of cows shortly before drying off, as well as the time and direct costs, may render this proposal impractical for most herds.
      2. The California mastitis test (CMT) is a simple cow side test that is quick, and low cost and may be used to define if the cow is likely to be infected in one or more glands at a point in time. However, interpretation of the results is subjective and thus should be performed by the prescriber on all cows shortly before drying off, which may be impractical and prohibitive. Further, although CMT testing may be a feasible short-term/temporary solution, there is no opportunity without regular milk recording information to monitor the outcomes from the drying-off decision-making and dry period management, as well as general udder health management.

**Herd-level information to support responsible prescribing:**

In addition to individual-cow information, each of the following factors are also needed to support responsible prescribing, both for dry-cow and in-lactation antibiotics:

1. A bona fide relationship with the herdowner, which enables the vet to carry out clinical examinations and exercise clinical judgement. This forms the basis of a robust Client Patient Practice Relationship (CPPR).
2. A sophisticated understanding of the farm in general, including the herd, the people, the facilities and farm management (in general, during lactation and at drying off). As clearly articulated in Article 107(1) of 2019/6, antimicrobial medicinal products should not be applied routinely nor used to compensate for poor hygiene, inadequate animal husbandry or lack of care or to compensate for poor farm management. In the case of a new client, engagement with the farmer, along with farm visits, clinical examinations, and appropriate ancillary testing will establish the necessary baseline of knowledge for responsible prescribing.
3. Knowledge/oversight of all antibiotics prescribed and used, and a holistic and coherent understanding of the rationale and strategy for antibiotic prescribing and use. This is only possible with a single prescriber (or single prescribing PVP practice) for each farm.
4. A thorough knowledge of the milk quality patterns of the farm, including temporal trends in bulk tank SCC and milk recording results, and access to accurate clinical mastitis records. The latter should also include treatment details, including outcomes.
5. A detailed knowledge of the mastitis pathogen challenge(s) on-farm, through regular milk culturing of individual cases (clinical and subclinical), and potentially also from the bulk tank (PCR testing). Antibiotic susceptibility testing (AST) should also be performed regularly, to guide appropriate antibiotic selection, and identify existing or emerging antibiotic resistance. This should include ongoing collection and analysis of the following milk samples, which may be frozen if necessary:
   1. A pre-treatment milk sample from all clinical cases, and
   2. Milk samples from cows with high SCC, ensuring a mixture of young and old cows, with evidence of both recent and chronic infections.
6. A detailed understanding of CellCheck resources, including the use of the CellCheck Dashboard as an investigative tool, to inform the farm assessment and prescribing decisions.
7. Records of mastitis events, treatments administered and related outcomes, preferably captured electronically, to facilitate monitoring and assessment, as well as improvement of treatment protocols.
8. A strong professional relationship between the prescriber and other professional farm service providers to ensure a holistic approach to milk quality and broader animal health and welfare.

With regards to Article 105 (3) of Regulation 2019/6, which states that a veterinary prescription shall be issued only after a clinical examination or any other proper assessment, it is the view of the CellCheck TWG that both animal and herd-level information will be required, as presented in detail above.

**National actions required**

The above individual animal and herd criteria reflect international best-practice for the prescribing of antibiotic therapy for mastitis control. As highlighted previously, this must be supported by a comprehensive range of national actions to facilitate optimal mastitis control and responsible prescribing across all Irish farms:

1. There needs to be a single prescriber (or single prescribing PVP practice) for each farm, with a sophisticated knowledge both of the farm (people, facilities, farm management including all aspects relevant to drying off) and of milk quality on the farm. This ensures knowledge/oversight of all antibiotics prescribed and used, but also a holistic and coherent understanding of the rationale and strategy for antibiotic prescribing and use.
2. There must be a comprehensive plan for the industry to increase awareness and education on responsible antibiotic use and any future legislative change, along with practical supports and resources to enable farmers to make the necessary transition. For example, the TASAH Dry Cow Consult currently supports farmers that meet the herd-level criteria recommended for adopting a selective drying off strategy (approx. 25% of the dairy population). However, for the remaining herds, appropriate support mechanisms should be developed and delivered, enabling farmers to reduce the level of risk in their herds to safely adopt a selective drying off strategy. These supports may range from signposting towards necessary ‘best practices’ such as whole herd milk recording, to more intensive longer-term problem-solving services. The latter has previously been discussed within the industry, as a potential solution for herds with chronic and/or intractable mastitis issues i.e., a multi-disciplinary ‘standard support package’ to sustainably resolve SCC concerns on individual farms (see Appendix 3).
3. Detailed monitoring of on-farm antibiotic usage is needed, including objective measurement, systems for benchmarking and defined thresholds for further investigation. This is needed at multiple levels: nationally, at the level of the prescribing veterinary practice, and at farm-level. Electronic capture of prescribing data will be central to these efforts.
4. Restrictions and/or bans on the use of specific antibiotics within the dairy industry are needed, specifically highest priority critically important antibiotics (HP-CIAs). There is currently a worrying trend in Ireland of an annual increase in the proportion of dry-cow antibiotic tubes sold containing a HP-CIA.
5. There must be clear, meaningful industry-agreed targets that reflect the ambition of the industry and the changes that need to happen to maximise udder health while reducing the use of antibiotics in compliance with the new Veterinary Medicines Regulation.
6. SCC data adjustment and interpretation, as applied in Ireland, has had a substantial impact over many years on farm eligibility to supply raw milk for processing of dairy products. In practice, it has facilitated ongoing supply from Irish farms regardless of the quality of the collected milk. There is an urgent need for review of regulatory drivers for change. The criteria for herd eligibility to supply needs to be redrafted, including the corrective action required and the performance to be achieved when milk quality standards are not met, to require all farms to sustainably resolve milk quality issues.
7. There is a need to leverage ongoing advances in data management and analysis, using milk quality data including bulk tank SCC, milk recording and milk culture results, that are (or potentially could be) collected, to facilitate the development and improvement of tools to assist with national and farm-level decision-making.
8. There is a need to ensure a network of laboratories with sufficient capacity and expertise to deliver bacterial culture and antibiotic sensitivity testing, and for these data to be available to enable integrated reporting
9. Detailed supporting research is needed, including ongoing analysis of national bulk tank SCC, to facilitate science-informed discussion of SCC-related issues, including problem herds. To date, such analysis has not been possible, due to difficulties with data access.
10. Drawing on European best-practice, there is a need for detailed treatment guidelines for veterinarians to help guide the responsible use of antibiotics in different clinical scenarios.
11. There is an opportunity to leverage Bord Bia standards to facilitate improved milk quality. As currently written, the Bord Bia standards within the Sustainable Dairy Assurance Scheme (SDAS) do not exceed the legislative baseline with respect to milk quality. It would greatly facilitate progress if the Bord Bia standards were reframed to introduce an emphasis on the performance to be achieved (that is, output- and outcome-based standards). In support of the AgClimatise roadmap, a requirement for milk recording would deliver multiple benefits for farmers (including timely, objective information to support mastitis control and sDCT decision-making), as well as to the broader industry in terms of production efficiency and mitigation of greenhouse gas emissions.
12. Ongoing education and training for prescribers is needed, to maximise their effectiveness across a range of areas (problem herd investigation, CellCheck resources and tools including the CellCheck dashboard, prudent antibiotic stewardship etc)
13. There is a need for ongoing support for farm-level mastitis control, through farmer education (conducted as a collaborative effort by PVPs, Teagasc, milk processors and DAFM), farmer peer learning through discussion groups, supportive milk pricing structures, and technological developments (including farm-level dashboards, economic cost-calculator etc).
14. National efforts are needed to facilitate holistic approaches to milk quality, with prescribers working collaboratively with other professional farm service providers, including farm advisors and milking machine technicians.
15. A national discussion is needed of the multiple conflicting interests that veterinarians face when making prescribing decisions, including the professional obligations to alleviate suffering while ensuring prudent prescribing, in the context of financial dependency on clients and risk avoidance.

**Guidance for the prescribing PVP (dry-cow antibiotics) from 28 January 2022** (see Appendix 4)

|  | **Milk recording** | **No milk recording** |
| --- | --- | --- |
| **Lower risk herds**,  *(Those where there is objective evidence that mastitis is under good control and the prevalence of infection is consistently low. Examples in support could include unadjusted bulk milk SCC consistently below 200,000 cells/mL, a dry period new infection rate of less than 10% etc.)* | Prescribing decisions  Follow the current CellCheck Dry Cow Strategy.  Make prescribing decisions informed by:   - Individual animal information *(as above),* - Herd-level information *(as above)*, and - European Medicines Agency (EMA) guidelines^[[3]](#footnote-3)^. | Prescribing decisions  In the absence of milk recording data, the prescribing PVP should use the following to identify individual cows that have evidence of infection, and therefore require antibiotic treatment:   - individual milk culture results, or - individual CMT, as carried out by the prescriber.   Prescribing decisions should be made using this information, informed by:   - The current CellCheck Dry Cow Strategy, - Herd-level information *(as above)*, and - EMA guidelines. |
|  | Mastitis control decisions  Provide professional support to maintain optimal mastitis control.  At the time of dry-cow prescribing:   - conduct a review of treatment of in-lactation cases in the past season and - develop/agree a standard operating procedure for the treatment of in-lactation cases in the following season. | Mastitis control decisions  The farmer should be required to immediately commence whole herd milk recording, within 6 months (or other nationally defined, time-limited period).  Provide professional support to maintain optimal mastitis control.  At the time of dry-cow prescribing:   - conduct a review of treatment of in-lactation cases in the past season, and - develop/agree a standard operating procedure for the treatment of in-lactation cases in the following season. |
| **Higher risk herds**  *(All other herds)* | Prescribing decisions  Follow the current CellCheck Dry Cow Strategy, with consideration to reduce the individual cow SCC threshold for antibiotic treatment from 100,000 cells/mL to 50,000 cells/mL.  Make prescribing decisions informed by:   - Individual animal information *(as above),* - Herd-level information *(as above),* and - EMA guidelines.   Where the risk of new infection over the dry period is unacceptable, the prescribing PVP may consider that prophylactic use of dry-cow antibiotic is justified in order to protect cow welfare. In these exceptional cases, VCI may consider approval of such prescribing for a single drying off season, on condition that each of the mastitis control decisions (below) are subsequently followed. | Prescribing decisions  In the absence of milk recording data, the prescribing PVP should use the following to identify individual cows that have evidence of infection, and therefore require antibiotic treatment:   - Individual milk culture results, or - Individual CMT, as carried out by the prescriber.   Prescribing decisions should be made using this information, informed by:   - The current CellCheck Dry Cow Strategy, - Herd-level information *(as above)*, and - EMA guidelines.   Where the risk of new infection over the dry period is unacceptable, the prescribing PVP may consider that prophylactic use of dry-cow antibiotic is justified in order to protect cow welfare. In these exceptional cases, VCI may consider approval of such prescribing for a single drying off season, on condition that each of the mastitis control decisions (below) are subsequently followed. |
|  | Mastitis control decisions  The farmer should engage with their PVP and associated professionals to sustainably resolve constraints to effective mastitis control. Each of the following will be needed:   - A detailed understanding of the factors (including cause(s) and driver(s)) contributing to suboptimal mastitis control based on a detailed on- and off-farm investigation, - A plan developed and agreed with the farmer to robustly and sustainably address each of these factors, including agreed actions and timelines and objective measures to monitor progress, and - Ongoing and regular assessment and review.   The proposed ‘standard support package’ (see Appendix 3) could facilitate the achievement of these objectives.  At the time of dry-cow prescribing:   - conduct a review of treatment of in-lactation cases in the past season, and - develop/agree a standard operating procedure for the treatment of in-lactation cases in the following season. | Mastitis control decisions  The farmer should be required to immediately commence whole herd milk recording, within 6 months (or other nationally defined, time-limited period).  The farmer should engage with their PVP and associated professionals to sustainably resolve constraints to effective mastitis control. Each of the following will be needed:   - A detailed understanding of the factors (including cause(s) and driver(s)) contributing to suboptimal mastitis control based on a detailed on- and off-farm investigation, - A plan developed and agreed with the farmer to robustly and sustainably address each of these factors, including agreed actions and timelines and objective measures to monitor progress, and - Ongoing and regular assessment and review.   The proposed ‘standard support package’ (see Appendix 3) could facilitate the achievement of these objectives.  At the time of dry-cow prescribing:   - conduct a review of treatment of in-lactation cases in the past season, and - develop/agree a standard operating procedure for the treatment of in-lactation cases in the following season. |

**Guidance for the prescribing PVP (in-lactation antibiotics) from 28 January 2022**

| **Farm mastitis pathogen challenge(s)/antibiotic resistance patterns are known** | **Farm mastitis pathogen challenge(s)/antibiotic resistance patterns are not known** |
| --- | --- |
| Prescribing decisions  Confirm diagnosis of mastitis during lactation, by clinical examination or other proper assessment  Select appropriate antibiotic, based on both cow and farm factors:   - Cow factors such as clinical findings, lactation number and treatment history, and - Farm factors such as farm pathogen profile, antimicrobial susceptibility testing (AST) and previous treatment outcomes^[[4]](#footnote-4)^.   Choose an antibiotic from the lowest category possible on the EMA Antimicrobial Advice Ad Hoc Expert Group (AMEG) list^[[5]](#footnote-5)^ that has been shown to be effective, given knowledge of the farm mastitis pathogen challenge(s) and antibiotic resistance patterns. | Prescribing decisions  Confirm diagnosis of mastitis during lactation, by clinical examination or other proper assessment.  Choose an antibiotic from the *‘EMA Category D: Prudence’* category.  Antibiotics from ‘higher’ categories *(EMA Categories B: Restrict, C: Caution)* should only be considered with supporting milk culture and antibiotic susceptibility results and only when there are no antibiotics in a lower category that could be clinically effective. |
| Mastitis control decisions  Mastitis events, treatments administered, and related outcomes should be recorded by the farmer and made available to the PVP for analysis to assist with future treatment decisions.  In-lactation mastitis incidence should be monitored.  Develop/conduct an annual review of a mastitis treatment plan for in-lactation cases. | Mastitis control decisions  Mastitis events, treatments administered, and the related outcomes should be recorded by the farmer and made available to the PVP for analysis to assist with future treatment decisions.  In-lactation mastitis incidence should be monitored.  Instigate measures to gain a detailed knowledge of the mastitis pathogen challenge(s) and antibiotic resistance patterns on the farm. This should include ongoing collection and analysis of the following milk samples, which may be frozen if necessary:   - 1. A pre-treatment milk sample from all clinical cases, and   2. Milk samples from cows with high SCC, ensuring a mixture of young and old cows, with evidence of both recent and chronic infections.   Develop/conduct an annual review of a mastitis treatment plan for in-lactation cases. |

**References**

EMA (European Medicines Agency) and EFSA (European Food Safety Authority), 2017. EMA and EFSA Joint Scientific Opinion, RONAFA. EFSA J 15, 1. https://doi.org/10.2903/j.efsa.2017.4666

Devitt, C., McKenzie, K., More, S.J., Heanue, K., McCoy, F., 2013. Opportunities and constraints to improving milk quality in Ireland: enabling change through collective action. J Dairy Sci 96, 2661–2670. https://doi.org/10.3168/jds.2012-6001

McAloon, C.I., McCoy, F., More, S.J., 2021. Trends in estimated intramammary antimicrobial usage in the Irish dairy industry from 2003 to 2019. JDS Commun 2, 271–276. https://doi.org/10.3168/jdsc.2021-0081

More, S.J., 2020. European perspectives on efforts to reduce antimicrobial usage in food animal production. Irish Vet J 73, 2. https://doi.org/10.1186/s13620-019-0154-4

More, S.J., Clegg, T.A., Lynch, P.J., O’Grady, L., 2013. The effect of somatic cell count data adjustment and interpretation, as outlined in European Union legislation, on herd eligibility to supply raw milk for processing of dairy products. J Dairy Sci 96, 3671–3681. https://doi.org/10.3168/jds.2012-6182

More, S.J., Clegg, T.A., McCoy, F., 2017. The use of national-level data to describe trends in intramammary antimicrobial usage on Irish dairy farms from 2003 to 2015. J Dairy Sci 100, 6400–6413. https://doi.org/10.3168/jds.2016-12068

**Appendix 1. Examples of international best-practice**

Denmark and the Netherlands are selected as examples of international best-practice, noting key similarities with Ireland:

- All are EU member states
- All have important national dairy industries
- The value and mix of dairy exports is not dissimilar
- All have a focus on dairy product quality. Quality assurance programmes are in place (Arla’s Arlagården as one example in Denmark; Bord Bia Sustainable Dairy Assurance Scheme in Ireland; FrieslandCampina Foqus planet as one example in the Netherlands) and all supply commodity for the manufacture of infant formula

| **Table 1 (Appendix 1). Value of dairy commodities exported in 2019, by country** | | | |
| --- | --- | --- | --- |
| Dairy commodities | Value exported in 2019  (USD, billions)^a^ | | |
|  | Denmark | Ireland | Netherlands |
| Milk and cream |  |  |  |
| Not concentrated or sweetened | 0.22 | 0.08 | 0.83 |
| Concentrated or sweetened | 0.24 | 0.57 | 1.52 |
| Butter and other fats and oils derived from milk | 0.30 | 1.28 | 1.52 |
| Buttermilk | - | 0.07 | 0.11 |
| Whey | 0.23 | 0.41 | 0.18 |
| Cheese and curd | 1.61 | 1.17 | 4.11 |
| a. International Trade Centre (a joint agency of the World Trade Organization and the United Nations), [www.trademap.org](http://www.trademap.org) | | | |

The EU exported about 615,000 tonnes of infant formula in 2019 to third markets, including from:

- Netherlands (224,414 tonnes) [36.5%]
- France (183,665 tonnes) [29.9%]
- Ireland (146,559 tonnes; €900m) [23.8%]
- Germany (88,142 tonnes) [14.3%]
- Denmark (~ 38,000 tonnes) [6.2%]

The following provides a summary of measures introduced in Denmark, Ireland and the Netherlands to ensure prudent prescribing and to limit antimicrobial usage in farm animal production, including their date of introduction:

| **Table 2 (Appendix 1). Date of introduction of measures to limit antimicrobial usage in farm animal production in three EU member states: Denmark, Ireland and the Netherlands** | | | |
| --- | --- | --- | --- |
|  | Date of introduction | | |
|  | Denmark | Ireland | Netherlands |
| Ban on prophylactic (preventive) use of antimicrobials | 1995 | - | 2012 |
| Decoupling of antimicrobial sales and profits | 1995 |  | - |
| Initial restrictions on the on-farm use of antimicrobial agents linked with veterinary oversight, including prescribed farm visits | 1995^a^ | - | 2014 |
| Introduction of requirement for a one-to-one relationship between the farmer and the veterinarian | 1995 | - | 2014 |
| The first annual report of on-farm antimicrobial usage | 1996 | - | 2012 |
| The first treatment guidelines to support veterinary clinical decision-making | 1996 | - | 2012 |
| Mandatory reporting of farm prescribing/usage data to national database | 2000^b^ |  | 2012 |
| The first restrictions (by industry or government) on the use of HP-CIAs in food animals | 2003 | 2018 | 2012 |
| The first national target on reduction in antimicrobial usage | 2010 | - | 2009 |
| The introduction of farm-level benchmarking | 2010 | - | 2012 |
| Differential taxes on the sales of antimicrobials and other medicines for veterinary use | 2013 | - | - |
| The introduction of prescriber benchmarking | - | - | 2012 |
| a. Voluntary from 1995, mandatory for larger farms from 2010  b. VetStat was established in 2000, reporting of veterinary prescribing data was mandatory from 2001 | | | |

Detailed information about Denmark and the Netherlands, including actions taken and observed changes in usage, are presented in the following pages.

| **Table 3 (Appendix 1). Actions taken in Denmark to reduce on-farm antimicrobial usage, by date of initial introduction** | | |
| --- | --- | --- |
| Year | References | Actions |
| 1993 | 1 | Restrictions on the use of the cascade rule (cheaper extemporaneously prepared medicines), imposing mandatory first priority to medicinal products approved for the relevant species, subsidiary approved for other species |
| 1995 | 1,2 | Initiation of DANMAP, the AMR surveillance programme |
| 1995 | 1,2,3 | New legislation limiting veterinary profit from direct sales of antimicrobials to 5-10% |
| 1995 | 1 | Treatment allowed only in diseased animals or animals in a well-defined incubation period (metaphylaxis) and prophylactic use became illegal |
| 1995 | 1,4 | Introduction of Veterinary Advisory Service Contracts (VASC) on a voluntary basis. The VASC results in frequent veterinary advisory visits, creating a one-on-one relationship between the farmer and the veterinarian, and provides the farmer with extended treatment possibilities. The most important aims of the VASC are to focus on advice and prevention of illness rather than treatment, to optimize the use of antimicrobials in order to minimize antimicrobial resistance and to improve animal welfare |
| 1995 | 1,2 | A limit on veterinary prescription to a maximum of 5 days of treatment in production animals. Exceptions only granted when a veterinary advisory service contract between the veterinarian and the farmer was signed. |
| 1995 | 1 | Mandatory registration by the veterinary practitioners of used, delivered and prescribed drugs to farmed animals. The information must be available for inspection by veterinary officials for 3 years) |
| 1996 | 1 | Pharmacies and the pharmaceutical industry prohibited from offering economic incentives to veterinarians or others for the purpose of increasing product sales |
| 1996 | 1 | Official guidelines for the choice of antimicrobial agents, providing specific recommendations for the selection of the appropriate antimicrobial agents for treatment of all common indications in major production animal species. Subsequently updated |
| 1996 | 1,2 | The first DANMAP annual report (subsequently published annually) |
| 2000 | 1,2 | Initiation of VetStat, the national database to monitor veterinary usage of antimicrobials. |
| 2001 | 1 | Mandatory reporting of veterinary prescribing to the VetStat database |
| 2002 | 1 | Fluoroquinolones intended for injection were restricted to use by the veterinary practitioner only |
| 2003 | 1 | Mandatory susceptibility testing in relation to use of fluoroquinolones for production animals, documenting the need. Notification of use of fluoroquinolones to the authorities is mandatory |
| 2005 | 1 | Action plan for reduction of the use of antimicrobials in food animals |
| 2006 | 1 | Regulation of antimicrobial agents for mastitis (recommending using simple penicillins) for herds in a health consultancy contract |
| 2010 | 1 | National target for 10% reduction in antimicrobial usage between 2010 and 2013 |
| 2010 | 1 | VASCs mandatory for larger herds, voluntary for smaller herds. The number of required advisory visits differs between species and age group |
| 2010 | 1 | Introduction of the ‘milk quality campaign’, seeking to reduce treatment of clinical mastitis by 50%, mainly through a reduction of treatment of subclinical mastitis, but also by increased monitoring of cell counts to determine the need for treatment |
| 2010 | 1 | Regulation of antimicrobial agents for mastitis (recommending using simple penicillins) for all herds. Emphasis from industry that narrow spectrum penicillins are used to treat mastitis caused by Gram-positive bacteria, unless sensitivity testing reveals resistance towards these antimicrobials. |
| 2010 | 1 | Introduction of the Yellow Card initiative for pigs, but also for cattle, targeting holdings with the highest antimicrobial consumption |
| 2013 | 1,2 | Implementation of differentiated taxes on the sales of antimicrobials and other medicines for veterinary use: vaccines (no tax), penicillins (simple and narrow spectrum) (0.8%), other veterinary antimicrobials (5.5%), critically important antimicrobials (3^rd^ and 4^th^ generation cephalosporins and fluoroquinolones) (10.8%), other veterinary medicines (not antimicrobials) (0.8%) |
| 2014 | 1 | Cattle industry target for 20% reduction in antimicrobial usage between 2012 and 2018 |
| 2014 | 1 | Voluntary industry ban on the use of 3^rd^ and 4^th^ generation cephalosporins, except as a last resort antimicrobial. |
| 2015 | 1 | Modification of the Yellow Card initiative, assigning different weights to different antimicrobial classes: fluoroquinolones, cephalosporins and colistin (added in 2017) were given the highest multiplication factor of 10. Tetracyclines were given a multiplication factor of 1.2, which was adjusted to a factor of 1.5 in 2017 |
| 2017 | 1 | Renewed industry strategy for a 20% reduction in use of antimicrobials for treatment of mastitis and other cattle diseases as well as lowering geometric mean bulk tank cell counts to 150,000 by the year 2020. In addition, the dairy industry promoted the use of dry-cow therapy and mastitis treatment using simple penicillins |
| 2018 | 1 | Establishment of an Advisory Committee on Veterinary Medicine with the objective to provide evidence-based professional advice for the Minister of Environment and Food in relation to the use of veterinary medicine and to tackle related issues proactively |
| 2019 | 1 | Industry ban on the use of 3^rd^ and 4^th^ generation cephalosporins in all cattle |
| 1. DANMAP annual reports. <https://www.danmap.org/reports>  2. EMA (European Medicines Agency) and EFSA (European Food Safety Authority), 2017. EMA and EFSA Joint Scientiﬁc Opinion on measures to reduce the need to use antimicrobial agents in animal husbandry in the European Union, and the resulting impacts on food safety (RONAFA). [EMA/CVMP/570771/2015]. EFSA J 15, 1. <https://doi.org/10.2903/j.efsa.2017.4666>  3. Wielinga, P.R., Jensen, V.F., Aarestrup, F.M., Schlundt, J., 2014. Evidence-based policy for controlling antimicrobial resistance in the food chain in Denmark. Food Control 40, 185–192. <https://doi.org/10.1016/j.foodcont.2013.11.047>  4. Animal Health in Denmark, 2018. Ministry of Environment and Food in Denmark. Danish Veterinary and Food Administration. <https://www.foedevarestyrelsen.dk/Publikationer/Alle%20publikationer/Animal_health_in_Denmark_2018.pdf> | | |


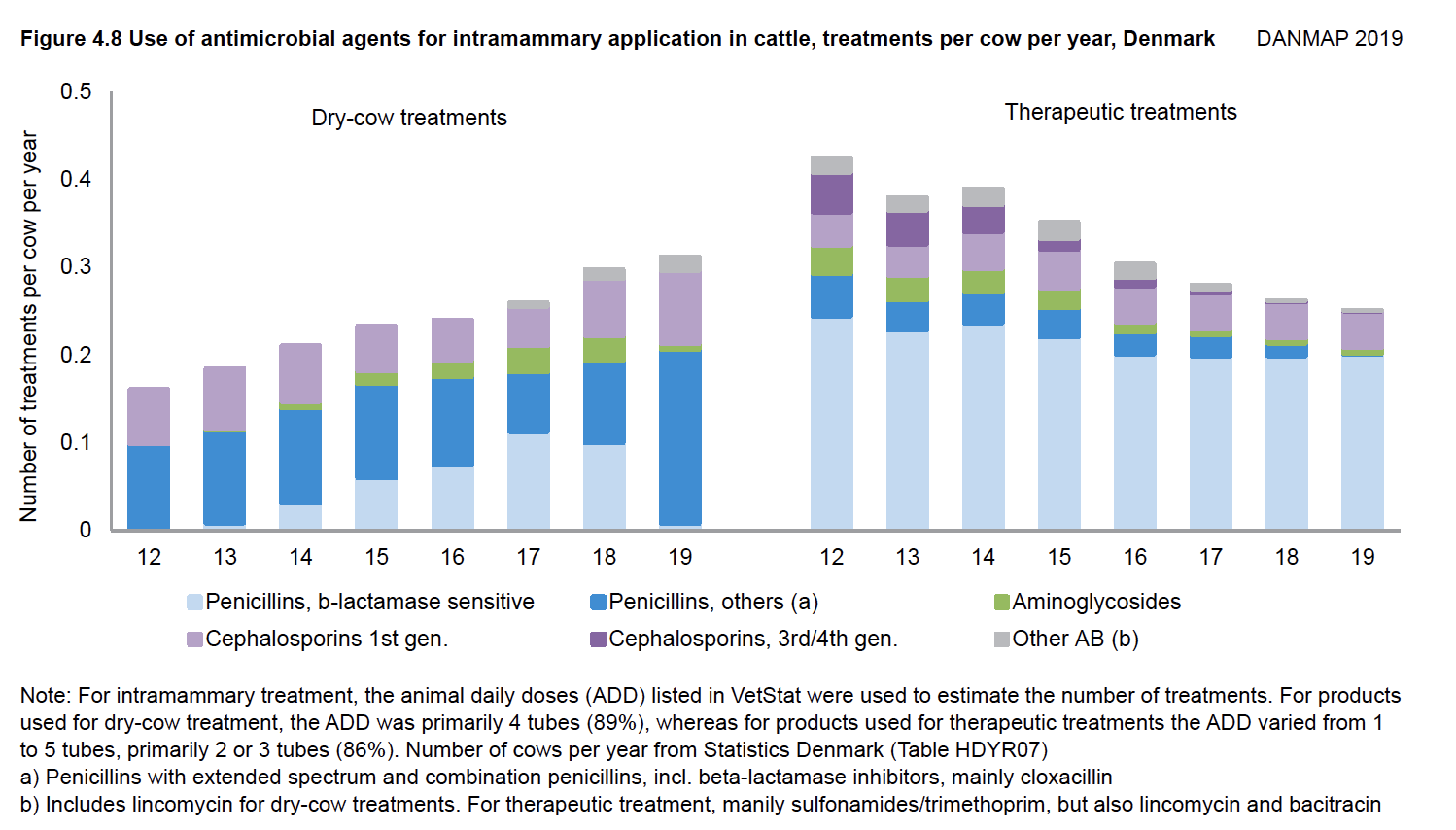

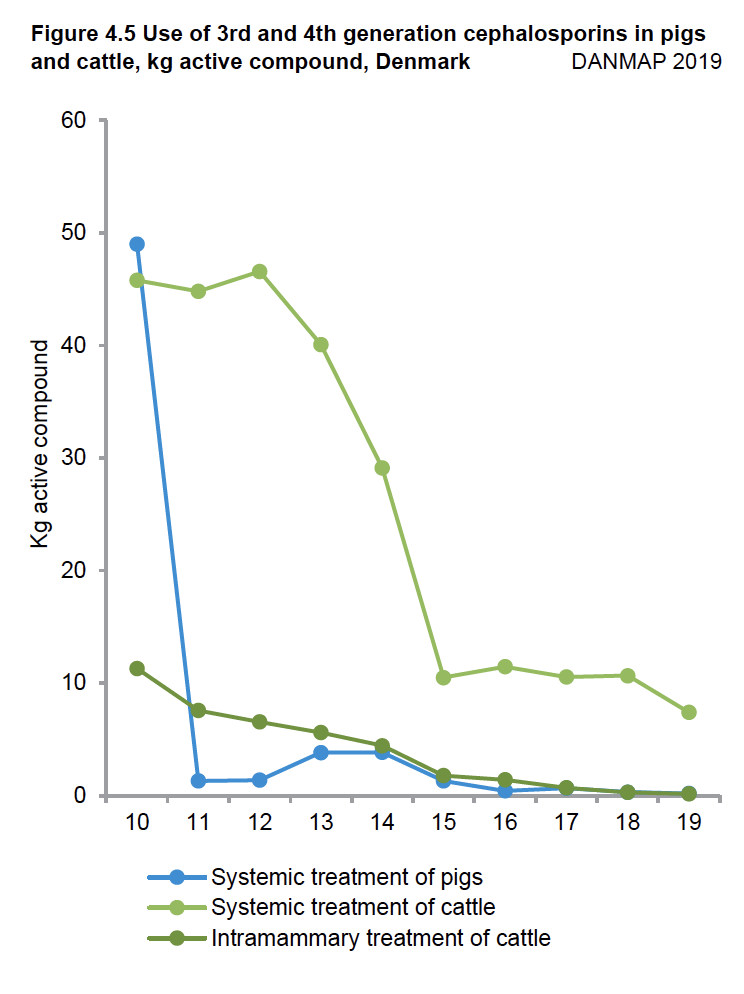


**Observed changes in on-farm antimicrobial usage in Denmark.**

From DANMAP 2019. Use of antimicrobial agents and occurrence of antimicrobial resistance in bacteria from food animals. <https://www.danmap.org/reports>

| **Table 4 (Appendix 1). Actions taken in the Netherlands to reduce on-farm antimicrobial usage, by date of initial introduction** | | |
| --- | --- | --- |
| Year | References | Actions |
| 2005 | 1 | Launch of the Dutch national udder health programme |
| 2008 | 2 | Stakeholder commitment to action plans of the Taskforce Antibiotic Resistance in Animal Husbandry, which committed to detailed monitoring of antimicrobial use at herd level, the monitoring of antimicrobial resistance, a clear separation of responsibilities for veterinarians and farmers in antibiotic prescriptions and the introduction of Farm Treatment Plans and Farm Health Plans |
| 2009 | 2,3 | Compulsory national targets for reduction in veterinary antimicrobial usage, of 20% by 2011 and by 50% in 2013, compared to 2009 |
| 2010 | 2 | Establishment of the Netherlands Veterinary Medicines Authority (SDa), with responsibility to collect and report reliable antimicrobial usage and prescribing data from all farms and veterinarians, and the setting of annual targets for usage in different livestock sectors, including benchmark indicators for farmers and veterinarians |
| 2010 | 2,4 | Investigation, but rejection, of the concept of decoupling. Rather, it was agreed that the economic motives to prescribe would be substantially eliminated through the benchmarking of antimicrobial prescription and use, and introduction of strict 1-to-1 farmer-veterinarian relationships |
| 2011 | 2 | Reclassification of veterinary antimicrobials into 1^st^, 2^nd^ and 3^rd^ choice for using in existing treatment guidelines |
| 2012 | 1,2,5,6 | A ban on the prophylactic (preventive) use of all antimicrobials in animals. After this date, blanket dry-cow therapy was no longer permitted |
| 2012 | 2,3 | The use of 3^rd^ and 4^th^ generation cephalosporins and fluoroquinolones was prohibited in farm animals unless, following a herd examination, a veterinarian substantiates that no alternative drug is available for the treatment of the particular health problem |
| 2012 | 7 | The first SDa annual report (subsequently published annually) |
| 2012 | 3 | A requirement of all farmers to submit their drug usage data to a central database for benchmarking purposes |
| 2012 | 7 | The establishment of benchmarking of antimicrobial usage on livestock farms |
| 2013 | 2,4 | Development of disease-specific formularies (treatment guidelines) to support veterinarians in their clinical decision-making. These formularies are binding, with any deviations having to be well-justified or otherwise potentially subject to disciplinary action |
| 2013 | 7 | The establishment of benchmarking of antimicrobial prescribing by veterinarians, with prescribing patterns assessed by means of the Veterinary Benchmark Indicator (VBI) |
| 2013 | 2 | Compulsory national target for 70% reduction in veterinary antimicrobial usage in 2015 compared to 2009 |
| 2014 | 6 | Introduction of guidelines on the implementation of selective dry-cow therapy, including cow-level selection criteria to decide whether to apply antimicrobials at drying up |
| 2014 | 2,4 | Legislation requiring all veterinary antimicrobials to be administered by veterinarians. Farmers are only allowed to administer antimicrobials to their animals under specific conditions:   - A 1-to-1 relationship with their veterinarian - Mandatory periodic herd inspection by their veterinarian - Annual evaluation of the Farm Health Plan and Farm Treatment Plan   Under these conditions, farmers are allowed to have 1^st^ choice antimicrobials in stock for one treatment of 15% of susceptible animals. Exemptions have been made for a few 2^nd^ choice antibiotics that were regarded essential to treat animals for specific indications and where no 1^st^ choice alternatives are available |
| 2015 | 7 | Revision of the benchmarking method and ongoing adaptation of the action and signaling thresholds for different livestock sectors and for veterinarians |
| 2016 | 7 | Calculation of antimicrobial usage using DDD_vet_ (the technical unit used by the European Medicines Agency), to promote international transparency |
| 2016 | 8 | Revision of the veterinary formularies, including further restriction on the use of colistin in farm animals |
| 1. Santman-Berends, I.M.G.A., Heuvel, K.W.H. van den, Lam, T.J.G.M., Scherpenzeel, C.G.M., Schaik, G. van, 2021. Monitoring udder health on routinely collected census data: Evaluating the short- to mid-term consequences of implementing selective dry cow treatment. J Dairy Sci 104, 2280–2289. <https://doi.org/10.3168/jds.2020-18973>  2. Speksnijder, D.C., Mevius, D.J., Bruschke, C.J.M., Wagenaar, J.A., 2015. Reduction of Veterinary Antimicrobial Use in the Netherlands. The Dutch Success Model. Zoonoses Public Hlth 62, 79–87. https://doi.org/10.1111/zph.12167  3. Kuipers, A., Koops, W.J., Wemmenhove, H., 2016. Antibiotic use in dairy herds in the Netherlands from 2005 to 2012. J Dairy Sci 99, 1632–1648. <https://doi.org/10.3168/jds.2014-8428>  4. EMA (European Medicines Agency) and EFSA (European Food Safety Authority), 2017. EMA and EFSA Joint Scientiﬁc Opinion on measures to reduce the need to use antimicrobial agents in animal husbandry in the European Union, and the resulting impacts on food safety (RONAFA). [EMA/CVMP/570771/2015]. EFSA J 15, 1. <https://doi.org/10.2903/j.efsa.2017.4666>  5. Vanhoudt, A., Hees-Huijps, K. van, Knegsel, A.T.M. van, Sampimon, O.C., Vernooij, J.C.M., Nielen, M., Werven, T. van, 2018. Effects of reduced intramammary antimicrobial use during the dry period on udder health in Dutch dairy herds. J Dairy Sci 101, 3248–3260. <https://doi.org/10.3168/jds.2017-13555>  6. Scherpenzeel, C.G.M., Tijs, S.H.W., Uijl, I.E.M. den, Santman-Berends, I.M.G.A., Velthuis, A.G.J., Lam, T.J.G.M., 2016. Farmers’ attitude toward the introduction of selective dry cow therapy. J Dairy Sci 99, 8259–8266. <https://doi.org/10.3168/jds.2016-11349>  7. Usage of antibiotics in agricultural livestock in the Netherlands. Autoriteit Diergeneesmiddelen (Netherlands Veterinary Medicines Institute, SDa) annual reports. <https://www.autoriteitdiergeneesmiddelen.nl/en/publications/general-reports>  8. Speksnijder, D.C., 2017 Antibiotic use in farm animals: supporting behaviour change of veterinarians and farmers. PhD thesis, University of Utrecht. <http://dspace.library.uu.nl/bitstream/handle/1874/349700/Speksnijder.pdf?sequence=1&isAllowed=y>  9. Usage of antibiotics in agricultural livestock in the Netherlands. Autoriteit Diergeneesmiddelen (Netherlands Veterinary Medicines Institute, SDa) annual report, 2019. <https://www.autoriteitdiergeneesmiddelen.nl/en/publications/general-reports> | | |


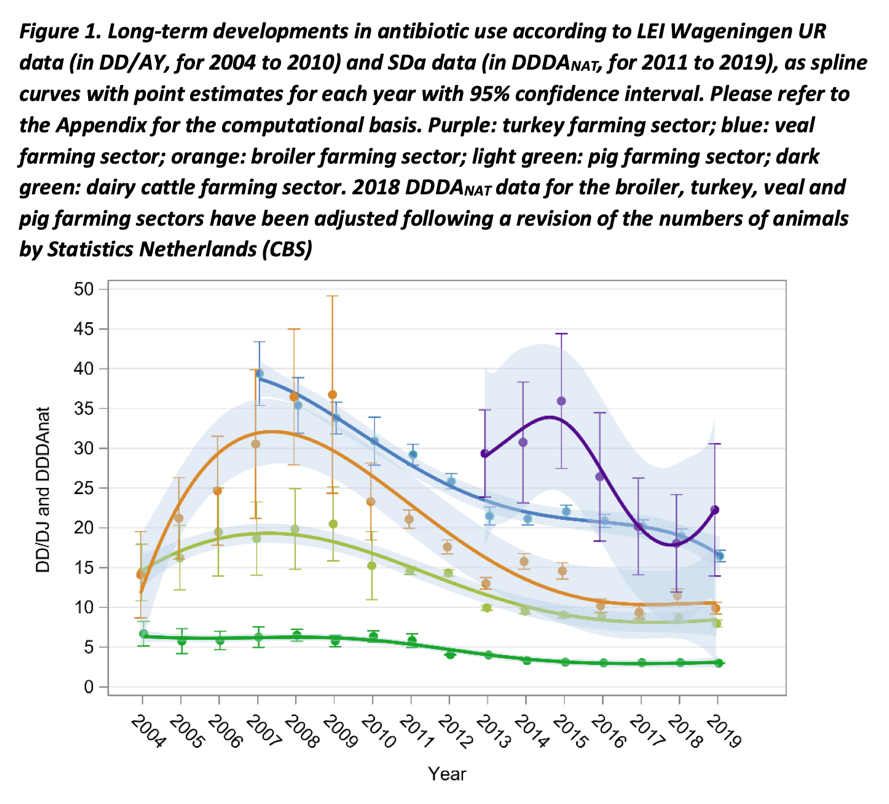


**Observed changes in on-farm antimicrobial usage in the Netherlands.**

From Usage of antibiotics in agricultural livestock in the Netherlands. Autoriteit Diergeneesmiddelen (Netherlands Veterinary Medicines Institute, SDa) annual report, 2019. <https://www.autoriteitdiergeneesmiddelen.nl/en/publications/general-reports>

**Appendix 2. CellCheck Dry Cow Strategy**

*Available online^[[6]](#footnote-6)^, updated October 2018*

- Antibiotics are essential medicines for humans and animals. The use of antibiotics on-farm is known to be one of the factors contributing to a rise in antimicrobial resistance, which in turn poses a risk to public (and animal) health in the future. It has been common practice to treat all quarters of all cows with an antibiotic tube at drying off, which is known as blanket dry-cow treatment. In order to help protect the ability of antibiotics to fight infection, the CellCheck Technical Working Group (TWG) supports the move away from blanket dry-cow treatment towards a selective dry-cow strategy, in a prudent manner, in appropriate herds.
- A selective dry-cow strategy involves administering internal teat sealant only to a selected proportion of suitable cows at drying off, with the remainder of the cows receiving both an antibiotic tube and an internal teat sealant.
- A selective dry-cow strategy is not without risk, hence the importance of these recommendations when making farm-level decisions.
- A selective dry-cow strategy may be considered in herds:

1. Where there is good evidence of a low prevalence of infection, for example a bulk milk SCC consistently below 200,000 cells/mL, a dry period new infection rate of less than 10% etc., and
2. Where good practices and high levels of hygiene can be achieved at drying off, throughout the dry period and at calving*, and
3. Where regular milk recording is carried out, with at least one recording in the last month prior to drying off, and
4. Where the herd keeper is willing to engage with their veterinary practitioner in decision-making around their dry-cow treatment programme.

- Within these herds:
  - Cows with an SCC consistently below 100,000 cells/mL**** throughout the lactation and with no history of clinical mastitis, or teat-end abnormality may be considered suitable for internal teat sealant only at drying off, provided high levels of hygiene can be achieved during administration.
  - In all other cows, the TWG recommends using an internal teat sealant as well as an antibiotic tube.

**For more details, see CellCheck Farm Guidelines for Mastitis Control-Guidelines 1, 17-19 and Management Notes D, E and L.*

*** There is ongoing Irish and international research to determine appropriate SCC (or other) thresholds, to select cows suitable for internal teat sealant only. As yet, however, appropriate thresholds have not been clearly defined. This is reflected in the use of differing cow thresholds in different countries. These AHI recommendations will be reviewed in the future on the basis of new research conclusions.*

**Appendix 3. Proposal for a standard support package to sustainably resolve SCC concerns on individual farms**

The CellCheck Implementation Group previously identified a need for a coordinated, standardised support package for herds with chronic mastitis problems (2017). The TWG was then tasked to develop an outline proposal of the technical supports required for intervention to be effective. This proposal for a “standard support package” outlined the tools, resources, timeframe and professional input likely to be required to effectively resolve a mastitis issue, in order to manage expectations.

These included, at a minimum:

- Prior participation in a CellCheck Farmer Workshop
- 2-year commitment to the support package
- Multi-disciplinary involvement (co-op advisor/farm advisor/milking machine technician/PVP), with a lead investigator
- Milking machine service twice a year
- Milk recording (the TWG recommends recording every 4-6 weeks, with a minimum of 6 recordings throughout the lactation)

The TWG also carried out a basic cost-benefit analysis, based on anticipated related costs and likely returns. The estimated return on investment over the 2-year time frame of the package was 2:1 for an 80-cow dairy herd (based on 2018 prices).

**Appendix 4. Dry-cow antibiotic prescribing guidelines**

**Dry-cow Antibiotic Prescribing Guidelines for the single prescribing PVP practice**

Do you have a Client Patient Practice Relationship (CPPR) with this farm?

**NO**

Do not prescribe

**YES**

Apply to VCI to allow blanket prescription for one season only, on condition that the farmer commences milk recording immediately and engages with their PVP and associated professionals to sustainably resolve constraints to effective mastitis control.

**NO**

Is individual animal information available?

**YES**

Prescribe for individual animals, following the CellCheck Dry Cow Strategy

1. Milk recording from all lactating cows using milk meters or eDIY equipment provides a representative milk sample from all 4 quarters, and thus are repeatable and reliable. Conversely, a sample taken manually from each cow and sent to a lab for SCC measurement does not provide a representative composite sample and is not recommended by the CellCheck TWG. [↑](#footnote-ref-1)
2. Supporting CellCheck resources will need to be developed, highlighting the value and return from milk recording data [↑](#footnote-ref-2)
3. <https://www.ema.europa.eu/en/documents/report/infographic-categorisation-antibiotics-use-animals-prudent-responsible-use_en.pdf> The full report is available at <https://www.ema.europa.eu/en/documents/report/categorisation-antibiotics-european-union-answer-request-european-commission-updating-scientific_en.pdf> [↑](#footnote-ref-3)
4. Supporting CellCheck resources will be developed, including a review of mastitis treatment during lactation, economic and welfare decisions, pathogen characteristics, interpretation of AST results, treatment outcomes etc. [↑](#footnote-ref-4)
5. <https://www.ema.europa.eu/en/documents/report/infographic-categorisation-antibiotics-use-animals-prudent-responsible-use_en.pdf> [↑](#footnote-ref-5)
6. https://animalhealthireland.ie/assets/uploads/2021/11/4-CellCheck-Dry-Cow-Strategy-July-2019.pdf?dl=1 [↑](#footnote-ref-6)
